# A lightweight YOLO-TinyFuse model for small target detection of olive fruits

**DOI:** 10.3389/fpls.2026.1773377

**Published:** 2026-02-24

**Authors:** Xinyu Yang, Yichun Lin, Qiwen Xiao, Ziyao Liang, Luyao Ma, Yaxi ShuoGuo, Kugu Ade, Zhaoguo Tong, Yu Chen, Ying Cao

**Affiliations:** 1 College of Information Engineering, Sichuan Agricultural University, Ya’an, China; 2 College of Food Science, Sichuan Agricultural University, Ya’an, China; 3 College of Water Conservancy and Hydropower Engineering, Sichuan Agricultural University, Ya’an, China; 4 Panxi Crop Improvement Key Laboratory of Sichuan Province, Xichang University, Xichang, Sichuan, China; 5 School of Arts and Media, Sichuan Agricultural University, Ya’an, China

**Keywords:** BiFPN, lightweight model design, ModifiedNeck, Olea europaea, P2 layer, small object detection, YOLOv8

## Abstract

In response to the challenges posed by the large number of small targets, complex backgrounds and significant computational load involved in detecting olives, this study presents YOLO-TinyFuse, a lightweight detection model developed based on YOLOv8n. This model incorporates the P2 high-resolution feature layer, a ModifiedNeck cross-scale fusion structure (ModifiedNeck) and a bidirectional feature pyramid network (BiFPN) dynamic weighting module within a unified architecture. This architecture simultaneously preserves high-resolution feature representations, enhances bidirectional multi-scale interaction and optimises weighted feature aggregation. This synergistic design substantially improves the recognition of small objects while reducing model complexity further. Evaluations conducted on a multi-scenario olive phenotyping dataset demonstrate that YOLO-TinyFuse achieves an mAP50 of 92.3% and a Recall of 84.5%. This represents improvements of 2.6% and 3.2% respectively over YOLOv8n, while reducing the parameter count by 6.76%. These results confirm that the proposed model provides a deployable, computationally efficient, real-time solution for target recognition on mainstream edge computing platforms in automated olive harvesting scenarios, and offers a reusable, lightweight framework for agricultural small-object detection tasks requiring high performance and optimised computational efficiency.

## 1 Introduction

The olive is a high-value economic crop that occupies a pivotal position in the global agricultural economy (Jimenez-Lopez et al., 2020; Romani et al., 2019; Mazzocchi et al., 2019). Its fruits are the main source of high-quality edible oil and are widely used in the food, cosmetics and related industries, generating substantial value throughout the industrial chain (Cinardi et al., 2024; García-Serrano et al., 2019; Difonzo et al., 2021). Throughout the olive growth cycle, tasks such as fruit detection are critical components of Precision orchard management. Accurate and efficient detection enables growers to monitor fruit numbers in real time and provides a scientific basis for determining the optimal harvest period. Furthermore, reliable fruit detection is essential for the development of automated olive harvesting technologies, playing a vital role in promoting intelligent production and supporting the sustainable development of the olive industry. In the field of object detection, the definition of a small object must be based on general criteria as well as scene-specific characteristics. As summarised in mainstream survey literature (Lin et al., 2017), a small object is typically defined as having a pixel size smaller than 32 × 32, occupying less than 0.01% of the image area or presenting feature-map dimensions below 10 × 10. However, in the detection of olives, small objects exhibit pronounced scene-specific properties: the mean pixel size of young fruits is often below 30 px, their phenotypic features are weak, and their grayscale contrast with the surrounding branches and leaves is often less than 15. These challenges are further compounded by the fine-grained feature loss induced by deep-network downsampling (Mamalis et al., 2023; Osco-Mamani et al., 2025), collectively increasing the difficulty of reliable detection. Considering these characteristics, olive fruit detection faces four primary challenges. First, small object features are highly prone to loss, as young fruits contain limited fine detail information that conventional models struggle to capture effectively (Li et al., 2023b). Secondly, substantial background interference occurs (Hou et al., 2022), with branch-leaf occlusion rates ranging from 20% to 80%, fruit clusters containing two to six overlapping fruits and frequent extreme illumination fluctuations, all of which reduce detection robustness. Thirdly, there is considerable variation in object scale, with fruit size differing by more than a factor of five from early developmental stages to maturity, which exceeds the adaptation capacity of many traditional models (Osco-Mamani et al., 2025). Fourthly, deployment conditions are constrained as mountainous orchards often rely on unmanned aerial vehicles (UAVs) and handheld edge devices. This imposes strict requirements on multi-scale feature extraction, cross-layer fusion and lightweight deployment efficiency (Zhu et al., 2025a; Ji et al., 2025).

Traditional approaches based on manual expertise or conventional image-processing techniques clearly have limitations in terms of both detection accuracy and cross-scene generalisation. Deep learning has therefore emerged as the dominant solution paradigm, giving rise to three principal technical pathways, each with inherent constraints in terms of adapting to different scenarios. Early convolutional neural networks (CNN) laid the groundwork for hierarchical phenotypic feature extraction, while the YOLO family has become the go-to choice for field applications thanks to its end-to-end architecture and real-time detection capability. More recently, Transformer-based models such as DETR have shown great promise due to their strong semantic association modelling capabilities. However, all three approaches still face practical limitations relating to environmental robustness, lightweight model design and real-time computational efficiency, which restrict their deployment in complex agricultural scenarios.

Firstly, convolutional neural networks (CNN) progressively refine fruit features through stacked convolution and pooling operations, and were among the earliest deep learning approaches applied to the detection of small objects in agriculture. However, existing CNN-based methods still face two fundamental limitations: First, insufficient adaptability to highly variable environmental and scene conditions; second, limited effectiveness in detecting small objects, particularly when fine-grained phenotypic features are weak or are lost easily during downsampling.

Cano Marchal et al. (2021) proposed an infrared-based automated system for detecting defects in olives. This system integrates an iterative active-contour algorithm with a decision-tree classifier and achieves a detection rate of 81.22%. However, the system lacks illumination-adaptive regulation and therefore cannot autonomously adapt to complex outdoor environments; manual parameter readjustment is required under field conditions with variable lighting. Beltrán et al. (2023) developed an edible olive quality inspection system with an optimized topological design that achieved a detection success rate of 99.8%. Nevertheless, the samples used were limited to ideal conditions, such as white trays, natural indoor lighting and fixed imaging distances, without accounting for practical challenges such as branch-leaf occlusion or uneven illumination. Consequently, the system has not been validated for robustness under real-world field conditions.

Jing et al. (2024) proposed MRD-YOLO, a lightweight detection framework which adopts YOLOv8n as the baseline. It achieves model compression by reducing redundant computations in the backbone, enhancing multi-scale feature fusion efficiency and incorporating a lightweight attention mechanism. The model achieved an mAP50 of 97.4% on a melon dataset constructed in-house. However, as its training samples primarily consisted of clear, medium-sized fruits, the model has limited capacity for effective phenotypic feature extraction from low-resolution, blurred, or extremely small fruits commonly encountered under field conditions. Consequently, the model remains susceptible to missed detections in complex agricultural environments.

Secondly, the YOLO family is characterised by its single-stage, end-to-end detection architecture. This achieves an effective balance between processing speed and accuracy, making it the mainstream solution for real-time detection of small objects in orchards. However, existing YOLO-based improvement strategies still have limitations in terms of lightweight adaptation and robustness under complex field conditions.

Osco-Mamani et al. (2025) used the YOLOv8m model to detect olive fruit and achieved an mAP50 of 94.96%. However, with 25.9 million parameters, the model is not suitable for agricultural field scenarios that require lightweight architectures and optimised computational efficiency. Using a public RGB dataset of rice planthoppers, Ji et al. (2025) proposed the SwinTYOLOv8n-p2 model, which integrates a Swin Transformer with YOLOv8n-p2 and incorporates SCConv to enhance the C2f module. This results in an mAP50 of 86.8%. Nevertheless, with 65.2 million parameters and a computational cost of 307.4 GFLOPs, the model exceeds the capacity of resource-constrained edge devices such as UAVs and robotic harvesting platforms. Similarly, Sapkota et al. (2024) introduced the YOLOv9 Gelan-e and Gelan-base models, which achieved an mAP50 of 93.5%. However, their inference latency is approximately eight times that of YOLOv8n, rendering them unsuitable for real-time monitoring in orchard environments.

Fu et al. (2024) proposed the YOLOv5-AT model to overcome the challenges posed by small dataset sizes and the high colour similarity between green fruits and the surrounding foliage. Following architectural optimisation, the model achieved an mAP50 of 84.6%. However, under complex field conditions, such as dense branch-leaf occlusion and severe illumination fluctuations, the model’s ability to discriminate fine-grained phenotypic features declines markedly, resulting in substantial degradation of detection performance. Zhu et al. (2025a) improved an olive fruit maturity detection algorithm based on YOLOv11n by replacing the backbone with an EfficientNet-B0 and integrating LSKA and BiFPN modules to improve lightweight adaptability. Nevertheless, when exposed to extreme orchard conditions, including midday overexposure, reflective water accumulation on leaves after rainfall and complete fruit occlusion by branches, the model’s missed detection rate increased sharply to over 15%, indicating insufficient robustness for reliable deployment.

Powered by the Transformer self-attention mechanism, DETR can capture long-range semantic dependencies between fruits and their background. This offers a new research pathway for detecting small objects in agriculture in complex environments. However, current DETR-based approaches still struggle to meet real-time field requirements due to limitations in inference efficiency and insufficient lightweight adaptability. Wang et al. (2021) introduced the SwinGD model, which enhances feature association capabilities through an optimised Transformer architecture, achieving an mAP50 of 94% for grape cluster detection in complex vineyard conditions. Nevertheless, the model relies heavily on deep semantic computation, resulting in reduced inference speed and limited computational efficiency. Consequently, it remains unsuitable for agricultural automation scenarios such as UAV-based real-time monitoring or synchronised detection in robotic harvesting systems, thereby constraining its practical deployment in orchard environments.

In summary, this study proposes an enhanced model, YOLO-TinyFuse, developed on the YOLOv8n baseline, to address the major technical bottlenecks in field-based olive-fruit detection. These include the loss of fine-grained small-object features during deep downsampling, the low efficiency of feature fusion under complex backgrounds, and the computational and endurance constraints of edge devices. This model is the first to integrate the ModifiedNeck structure (Subedi, 2024), the BiFPN bidirectional weighted feature fusion module (Arısoy and Uysal, 2025; Wu, 2025; Li et al., 2024) and the P2 high-resolution feature layer (Zhu et al., 2025b; Ma et al., 2023). Specifically, the ModifiedNeck structure mitigates the issue of unidirectional information loss in traditional FPNs by introducing bidirectional pathways and channel unification strategies. The BiFPN module employs learnable weights to optimise cross-scale feature aggregation, while the P2 layer compensates for the absence of high-resolution representations, which are essential for the reliable detection of small objects. The synergistic interaction of these components establishes a comprehensive framework for phenotypic feature extraction, multi-scale fusion and optimised validation in YOLO-TinyFuse, effectively overcoming the key challenges inherent in olive fruit detection under real-world agricultural conditions.

This model innovatively integrates three core components: the P2 layer operates at a high resolution of 160×160 and preserves fine-grained details after 4× downsampling to enable reliable small object detection. The enhanced neck module effectively overcomes the inherent unidirectional information loss and redundancy issues of traditional FPN structures through bidirectional paths and channel unification. The bidirectional feature pyramid network module enhances the contribution of occlusion region features through dynamically weighted aggregation. This synergistic multi-module design achieves a 2.6% improvement in mAP50, a 3.2% increase in recall, and a 6.76% reduction in parameters, balancing detection performance, computational efficiency, and deployment costs.

To enhance model robustness under complex field conditions, a comprehensive scene-specific dataset was constructed covering three olive fruit development stages early, enlarged, and mature, diverse lighting conditions including sunny, cloudy, foggy, and rainy environments, and extensive occlusion levels such as 20-80% foliage coverage and 2–6 fruit overlaps per cluster, significantly boosting model generalization.

With a lightweight configuration of only 2.96 million parameters and an inference speed of 18.6 frames per second, YOLO-TinyFuse can be directly deployed on edge computing platforms like the Raspberry Pi 4B and drone systems, meeting end-to-end detection needs across the entire olive production chain. Furthermore, its technical framework exhibits strong reusability, efficiently adapting to other crops including wheat and cherries, small target objects such as bees, and highly occluded crops like mangoes and apples. This drives the evolution of agricultural phenotyping detection from single-crop customization toward multi-crop universal applicability.

## 2 Materials and methods

### 2.1 Data and preprocessing

#### 2.1.1 Dataset construction

The images in this dataset were collected in collaboration with the Beihe and Yuehua olive plantations within the Xichang Olive Industry Science and Technology Demonstration Zone in Sichuan, China, at 102.24°E and 27.74°N. The images were acquired using a Nikon D7200 camera at a resolution of 6000 x 4000 pixels in September 2024. The output resolution was set to 300 dpi with a colour depth of 24 bits and the sRGB colour mode. The camera was configured with an f/5.3 aperture, a 1/250 s exposure time and a 90 mm focal length to ensure clear visualisation of the key phenotypic features of the olives. These settings enabled effective separation of fruits from background vegetation under varying illumination conditions, while the controlled depth of field and precise focus highlighted fruit colour, texture, and morphological structure. This minimised detail loss caused by occlusion or rapid light fluctuations. Data collection encompassed three critical developmental stages-young fruit, enlargement and maturity, and included diverse illumination scenarios, such as midday sunlight, overcast conditions, and low-light evening environments. The dataset also captured representative orchard backgrounds, including dense branch-leaf occlusion, overlapping fruit clusters and single isolated fruits. This comprehensive, multi-scenario coverage avoids the limitations of generalization typically associated with single-condition datasets, providing a high-quality foundation for robust small-object detection and phenotypic feature extraction tasks in olive orchards.

**Figure 1:**
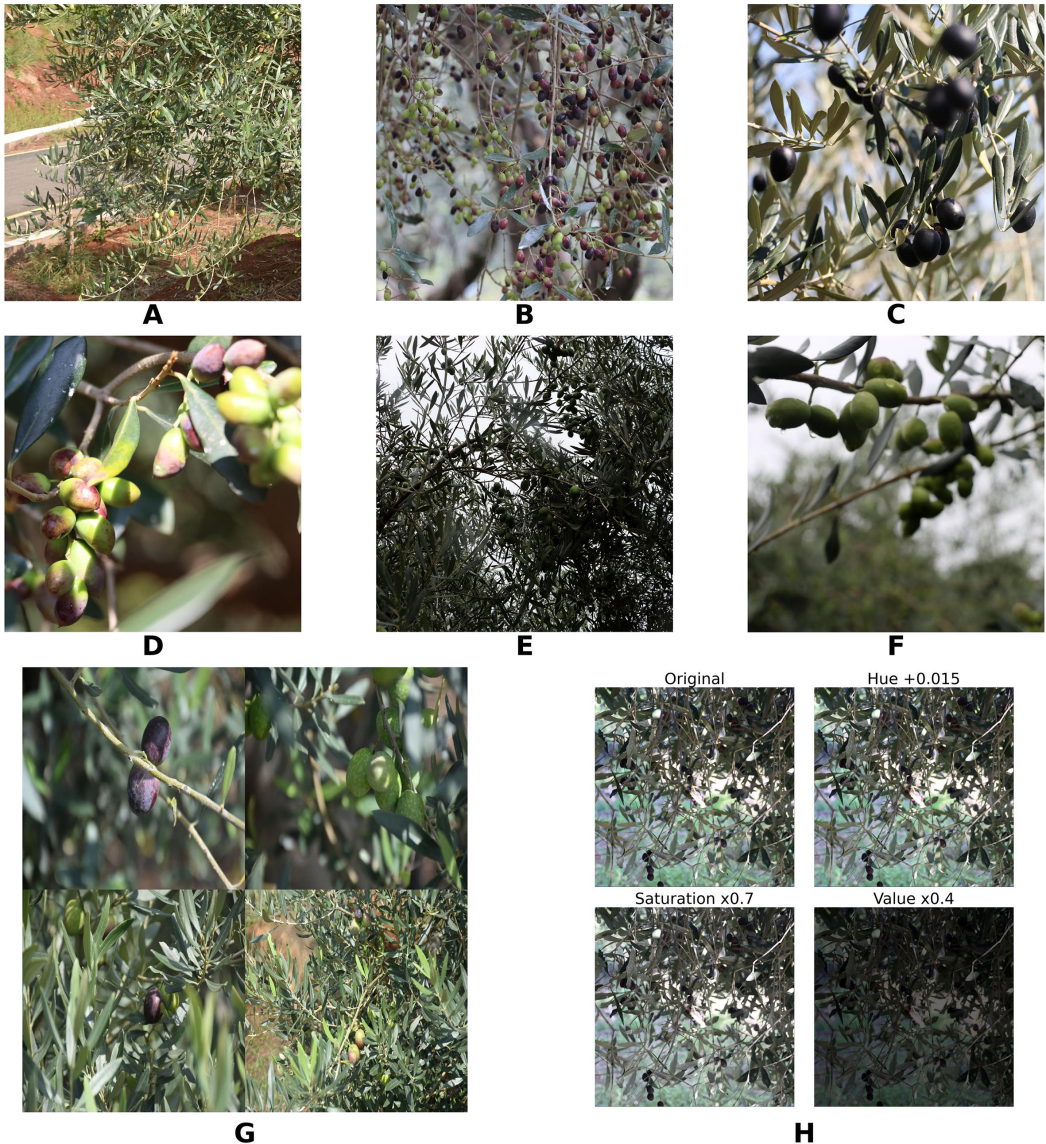
Representative samples and data processing results. **(A)** Young Fruit Stage. **(B)** Enlargement Stage. **(C)** Maturity Stage. **(D)** Sunlight. **(E)** Overcast. **(F)** Low-light evening. **(G)** Mosaic Enhanced. **(H)** Colour Space Adjustment.

The dataset was segmented using a stratified random sampling strategy. To prevent model overfitting caused by high inter-frame correlation, this strategy strictly adheres to the dual independence principle of “tree individuals + scenes.” Specifically, all olive trees collected from the Beihe and Yuehua plantations were first assigned unique identification numbers, grouping all images of each tree into independent “individual clusters”; Simultaneously, a three-dimensional stratification was applied based on “growth stage + light conditions + shading level “ to ensure consistent scene distribution across the training, validation, and test sets. During segmentation execution, independent individual groups were first randomly sampled at a 7:2:1 ratio. Images within each group underwent secondary sampling based on scene dimensions, ultimately retaining 12,259 high-quality images: 8,549 (69.7%) for the training set, 2,442 (19.9%) for the validation set, and 1,268 (10.4%) for the test set. This segmentation method ensures that images from the same plant do not span across datasets, effectively reducing inter-frame correlation and guaranteeing the authenticity and reliability of model generalization capability assessment. **Figure 1** shows representative samples from the dataset.

#### 2.1.2 Annotation protocol and quality control

Image annotation was conducted using the open-source LabelImg 1.8.6 tool, with annotations stored in.txt format. This ensures full compatibility with the YOLO family of detection algorithms and facilitates direct integration into subsequent model training workflows. Following annotation, a dual quality-control mechanism combining manual inspection and automated verification was implemented to ensure the reliability of the annotations. A 10% manual sampling rate was applied, paying particular attention to verifying the completeness and Precision of annotations for small objects. Automated validation scripts were used to check the accuracy of the bounding box coordinates and class labels. This two-tiered quality control procedure resulted in final annotations for the dataset, thereby meeting the rigorous quality requirements necessary for high-performance model training.

#### 2.1.3 Data preprocessing and augmentation strategies

To improve the robustness of the model in complex orchard environments while keeping computational costs under control, targeted pre-processing and augmentation procedures were applied to the training dataset. First, all images were uniformly resized to 640 × 640 pixels using bilinear interpolation and normalised within the RGB colour space to ensure consistent input characteristics across samples. A series of scene-adaptive augmentation operations were then implemented to increase the model’s resilience to variations in illumination, occlusion, and fruit morphology. These operations included Mosaic augmentation, horizontal flipping, geometric transformations, and HSV colour-space adjustments (Jing et al., 2024; Li et al., 2024; Zheng et al., 2025). It is important to note that Mixup augmentation and rotation-based transformations were intentionally excluded. Mixup can blend fruit and background pixels, which could degrade phenotypic feature extraction. Excessive rotation may also distort fruit morphology and introduce annotation misalignment. Both scenarios could adversely affect training stability and reduce detection accuracy. **Figure 1** shows representative outputs of the applied augmentation strategies.

### 2.2 Overview of the YOLO-TinyFuse architecture

**Figure 2:**
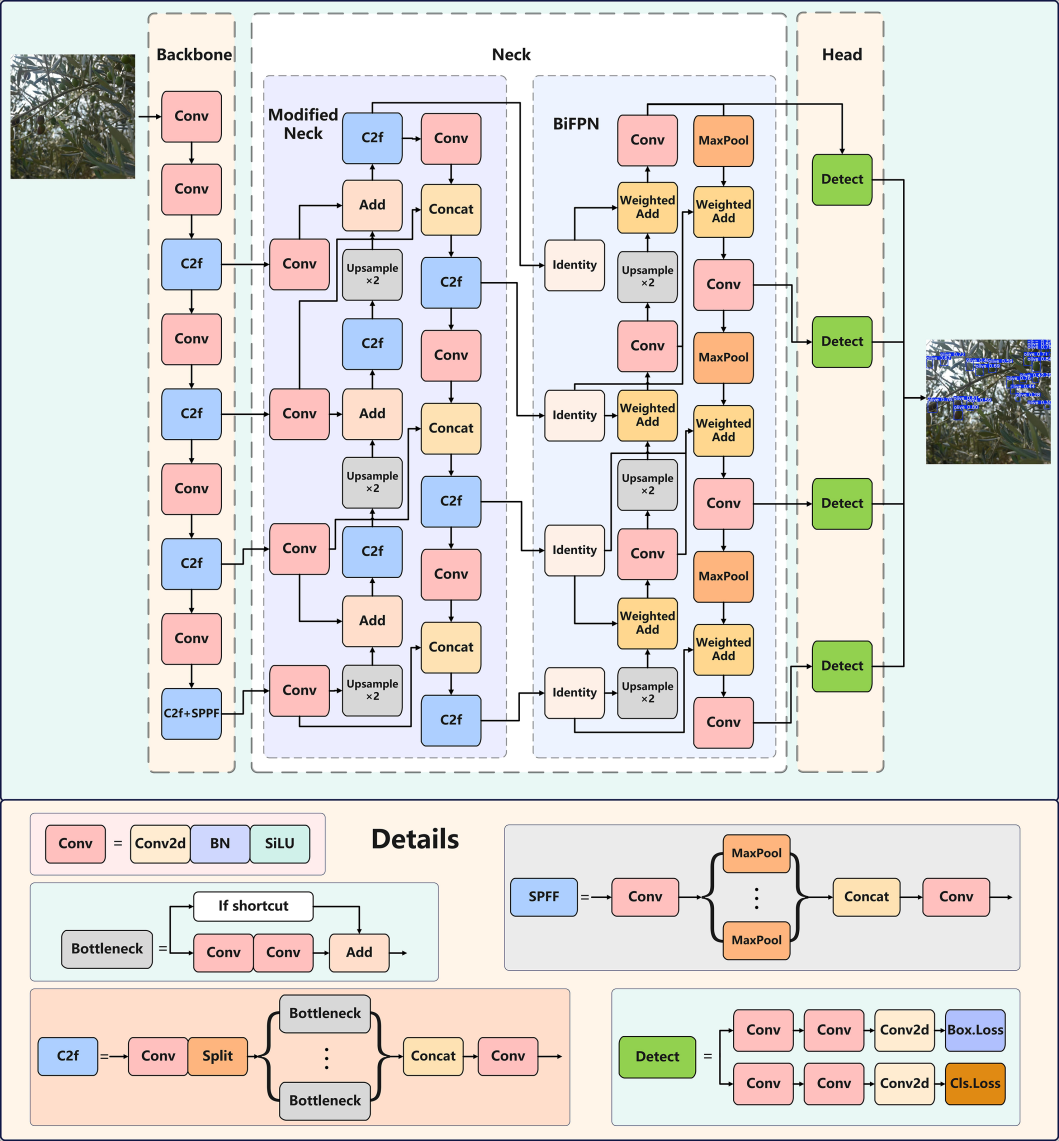
YOLO-TinyFuse framework and details. The backbone network adds a P2 high-resolution layer, paired with the C2f module to retain fine textures, and expands the receptive field through SPPF to generate P2-P5 multi-scale features. The neck module first passes through the ModifiedNeck, uses 1x1 convolution to unify the channels, and transmits information through bidirectional cross-scale fusion. Then, BiFPN is used to introduce learnable weights, combined with depth-wise separable convolution to amplify the contribution of each scale and optimize feature aggregation. Detection head: A P2-P5 four-scale decoupled head is adopted, where the classification and regression branches operate independently, and NMS is used to suppress redundant predictions, achieving accurate recognition of small, medium, and large targets. Arrows indicate downsampling upsampling and aggregation flows.

This study introduces an enhanced detection model, YOLO-TinyFuse, to address the limitations of the original YOLOv8n model, which framework is depicted in **Figure 2**. These limitations include a high missed-detection rate for small objects, low efficiency of feature fusion under complex orchard backgrounds, and substantial computational overhead. This new architecture incorporates three essential components for the first time: the ModifiedNeck, the BiFPN and the P2. YOLO-TinyFuse retains the three-stage Backbone-Neck-Head architecture typical of the YOLO series (Redmon et al., 2016; Ultralytics Team, 2023), while optimising the flow of feature information and the detection pipeline across three levels.

In the backbone, which is derived from the CSPDarknet structure of YOLOv8n, a new P2 feature layer has been introduced to retain more fine-grained details, which are essential for extracting the phenotypic features of small objects. Alongside the P3, P4 and P5 layers, the P2 layer forms a multi-scale feature pyramid that progressively captures information ranging from low-level textures to high-level semantic representations. These multi-scale features are then forwarded to the Neck.

The Neck is an improvement on the simplified FPN used in YOLOv8n, incorporating both the ModifiedNeck and BiFPN modules. The ModifiedNeck module performs preliminary cross-scale fusion through lateral connections, combining top-down and bottom-up information flows. The BiFPN module then conducts more refined feature aggregation based on this output, introducing learnable weighting mechanisms to achieve deep, scale-adaptive fusion and strengthen the expressiveness of the fused feature maps.

In the head, a dedicated P2 detection layer has been added for the specific purpose of detecting small objects, which substantially enhances the accuracy of detecting young olives. The P2, P3, P4 and P5 layers correspond to targets of different sizes. Their outputs are then merged and processed using non-maximum suppression (NMS) to generate the final predictions, which include bounding box coordinates, class labels and confidence scores.

Overall, YOLO-TinyFuse retains the real-time inference capabilities of the YOLO family, while also improving detection performance and model compactness. This dual optimisation makes the model highly suitable for practical field-scale olive-fruit detection in resource-constrained orchard environments.

#### 2.2.1 Backbone feature extraction: CSPDarknet

**Figure 3:**
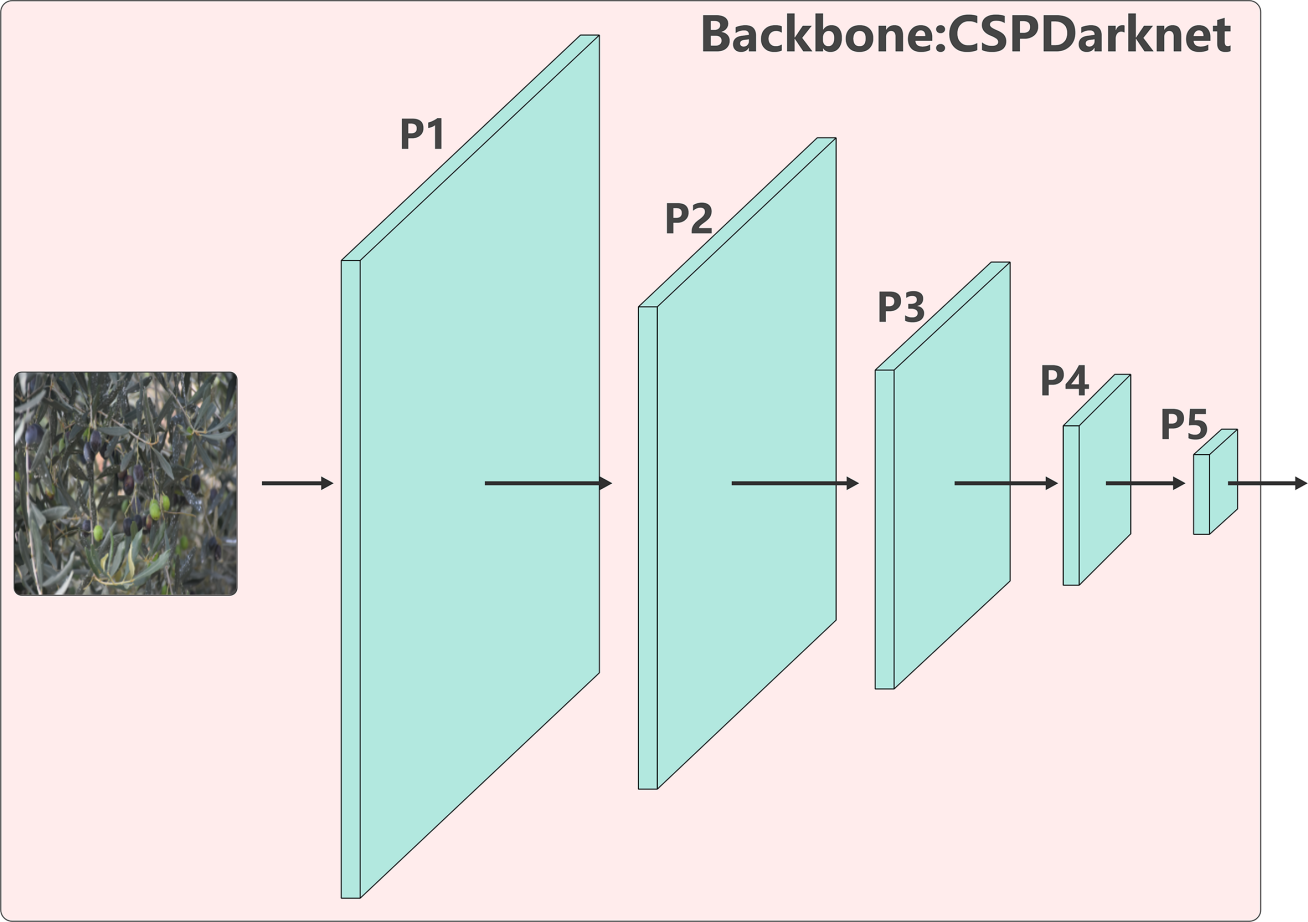
CSPDarknet structure. This streamlined backbone uses initial convolution to extract base textures. C2f blocks progressively yield P2-P5 multi-scale features. SPPF enlarges the receptive field. Downsampling ratios P2/4/8/16/32 correspond to detail and semantics suitable for different object sizes.

YOLO-TinyFuse uses a lightweight backbone based on the CSPDarknet architecture, which Structure is depicted in **Figure 3**. This architecture uses stacked multi-stage convolutional blocks and feature-aggregation modules to efficiently extract multi-scale representations from input images. Through progressive downsampling, the network constructs a hierarchical feature pyramid that preserves low-level textural details while simultaneously capturing high-level semantic information. This provides rich, multidimensional feature support for subsequent object-detection stages. The specific feature-extraction workflow is outlined as follows:

1. Initial feature mapping: The input is a 640 × 640 × 3 RGB image which is first processed by an initial convolutional layer comprising Conv + Batch Normalisation (BN) + Sigmoid Linear Unit (SiLU) activation. This expands the channel dimension from 3 to 16 and performs the first downsampling via stride-2 convolution, resulting in a feature map measuring 320 × 320 × 16.

2. Shallow Feature Extraction: After a stride-2 convolution operation increases the channel dimension to 32, the resulting feature map is fed into the first C2f module. This module employs a multi-branch parallel bottleneck architecture to refine the feature representation, ultimately generating the P2/4 feature layer (obtained through initial convolutional downsampling and refinement by the C2f module, with a resolution of 160×160 pixels and 32 channels, corresponding to 4x downsampling). This 4x downsampling design precisely matches the 640×640 input size: for olive fruits averaging less than 30px in size, a 20×20 px small target corresponds to 5×5 feature points, while an extremely small 10×10 px target still corresponds to 2.5×2.5 feature points. This effectively avoids the loss of fine-grained information compression caused by traditional 8x downsampling. Simultaneously, the P2 layer exhibits strong responsiveness to small target features, capturing core fine-grained, low-dimensional phenotypic characteristics such as texture and edges. The multi-branch parallel convolution structure of the C2f module further refines these feature representations, providing high-distinctiveness phenotypic information for small object detection. This successfully addresses the detection challenge posed by the low grayscale contrast (*<*15) between olive fruits and surrounding foliage.

3. Progressive Multi-Scale Feature Generation: The feature map is downsampled further via a stride-2 convolution to 80 × 80 × 64, then processed through a C2f module to produce the P3/8 feature layer (obtained after downsampling via stride-2 convolution on the P2/4 feature layer and refinement by the C2f module; resolution 80×80, 64 channels, corresponding to 8x downsampling; core feature representation tailored for medium-scale objects). This layer primarily targets medium-scale object representations. Repeating this sequence of downsampling and C2f refinement generates the P4/16 feature layer (obtained after downsampling via stride-2 convolution on the P3/8 feature layer and refinement through the C2f module; resolution 40×40, 128 channels; corresponds to 16x downsampling; core layer for representing mid-level semantic and morphological features of medium-to-large-scale objects) and the P5/32 feature layer (obtained after downsampling via stride-2 convolution on the P4/16 feature layer and refinement through the C2f module; resolution 20×20, 256 channels; corresponds to 32x downsampling; focuses on extracting high-dimensional global semantic features for large-scale targets; output fed into the SPPF module to enhance global feature representation). After the P5 layer, the C2f output is fed into the SPPF module (Jocher et al., 2020), which uses a kernel size of 5 and three consecutive max-pooling operations to perform multi-scale pooling. This process enriches receptive-field diversity and strengthens global semantic feature expression.

Finally, the backbone outputs four feature layers (P2, P3, P4 and P5) with spatial dimensions and channel depths of 160 × 160 × 32, 80 × 80 × 64, 40 × 40 × 128 and 20 × 20 × 256 respectively. These layers cover downsampling ratios ranging from 4× to 32×, enabling the learning of dedicated representations for small, medium, and large targets. Together, they provide a hierarchical, structured foundation of features for subsequent multi-scale fusion in the Neck module.

#### 2.2.2 Neck multi-scale feature fusion

This study introduces a two-stage fusion architecture composed of the ModifiedNeck and BiFPN modules to address the limitations of the simplified FPN structure in the original YOLOv8n, specifically insufficient information flow across scales and suboptimal weighting of multi-scale features. This architecture markedly enhances the efficiency and accuracy of cross-scale feature integration through a progressive fusion strategy, thereby improving the model’s capability for robust small-object detection in complex orchard environments.

##### 2.2.2.1 ModifiedNeck feature fusion module

**Figure 4:**
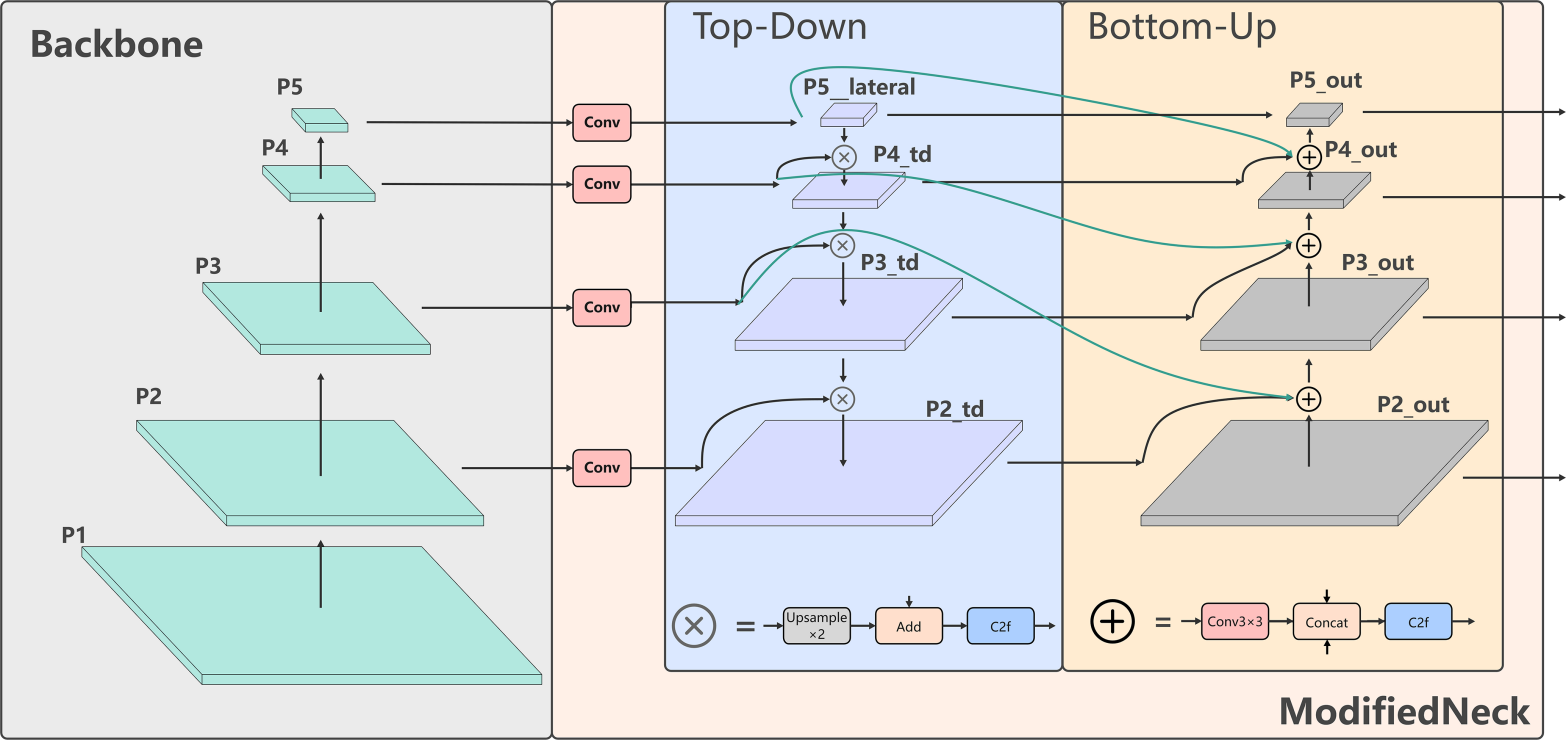
ModifiedNeck structure of the feature fusion module. P2 to P5 channels are unified to 64 via 1x1 convolution. Features are then fused by dual top-down and bottom-up paths. Upsampling passes semantics downward while downsampling feeds details upward. C2f refines the fused maps producing a unified P2 out to P5 out pyramid.

The design of the ModifiedNeck module focuses on bidirectional path interaction, channel unification and residual enhancement, which Structure is depicted in **Figure 4**. It receives four feature layers from the Backbone, which are processed through a channel unification operation to produce standardised feature maps (P2_out, P3_out, P4_out and P5_out) with a depth of 64 channels (Arısoy and Uysal, 2025). **Figure 4** illustrates the overall structure of the module, and its implementation involves four core steps, which are outlined below:

1. Lateral connections and channel unification: To mitigate fusion difficulties arising from substantial discrepancies in channel dimensions across feature layers, the channel depth of each input feature map is first standardised using a 1 × 1 convolution. This operation uniformly adjusts the P2, P3, P4 and P5 feature layers to 64 channels. This reduces the computational burden of subsequent fusion operations and ensures dimensional consistency across all feature representations. This process can be expressed mathematically in Equation 1:

(1) $$P_{i}^{\text{lateral}}={\text{Conv}}_{1\times 1}(P_{i}^{\text{backbone}})$$

Among them, $P_{i}^{\text{backbone}}$ denotes the i-th feature map produced by the Backbone (i=2,3,4,5); ${\text{Conv}}_{1\times 1}(\cdot )$ represents the 1 × 1 convolution operation; $P_{i}^{\text{lateral}}$ refers to the lateral feature map obtained after channel unification.

2. Top-Down path:P5 is first upsampled by a factor of two and then fused with the corresponding $p_{i}^{\text{lateral}}$ through element-wise addition. The fused features are subsequently refined using a C2f module, enabling effective downward propagation of semantic information. This process is formally expressed in Equation 2:

(2) $$P_{i}^{\text{td}}=\text{C}2\text{f}(P_{i}^{\text{lateral}}+\text{Upsample}(P_{i+1}^{\text{td}}))$$

Among them, Upsample(·) denotes the two-fold bilinear interpolation for upsampling; C2f(·) refers to the C2f-based feature enhancement module.

3. Bottom-Up pathway: starting from $P_{2}^{\text{td}}$, the feature map is downsampled using a stride-2 3×3 convolution and then concatenated with the corresponding $P_{i+1}^{\text{lateral}}$, resulting in a combined feature map with 128 channels. This fused representation is then enhanced through a C2f module and reduced back to 64 channels, enabling effective upward feedback of fine-grained information. The process is mathematically expressedtd in Equation 3:

(3) $$P_{i}^{\text{out}}=\text{C}2\text{f}(\text{Concat}(P_{i+1}^{\text{lateral}},\text{Downsample}(P_{i+1}^{\text{out}})))$$

Among these, Downsample(·) denotes the stride-2–3 x 3 convolution for downsampling; Concat(·) refers to the feature concatenation operation; C2f(·) indicates the C2f enhancement and fusion module.

4. Lightweight optimisation: After the bidirectional fusion process is complete, each of the four feature layers is passed through an output projection stage for final refinement. This produces a unified, 64-channel, multi-scale feature pyramid consisting of P2_out, P3_out, P4_out and P5_out.

These optimisations substantially improve the efficiency of feature fusion and enhance the representational capacity of features across different spatial scales through the ModifiedNeck module.

##### 2.2.2.2 BiFPN weighted fusion mechanism

**Figure 5:**
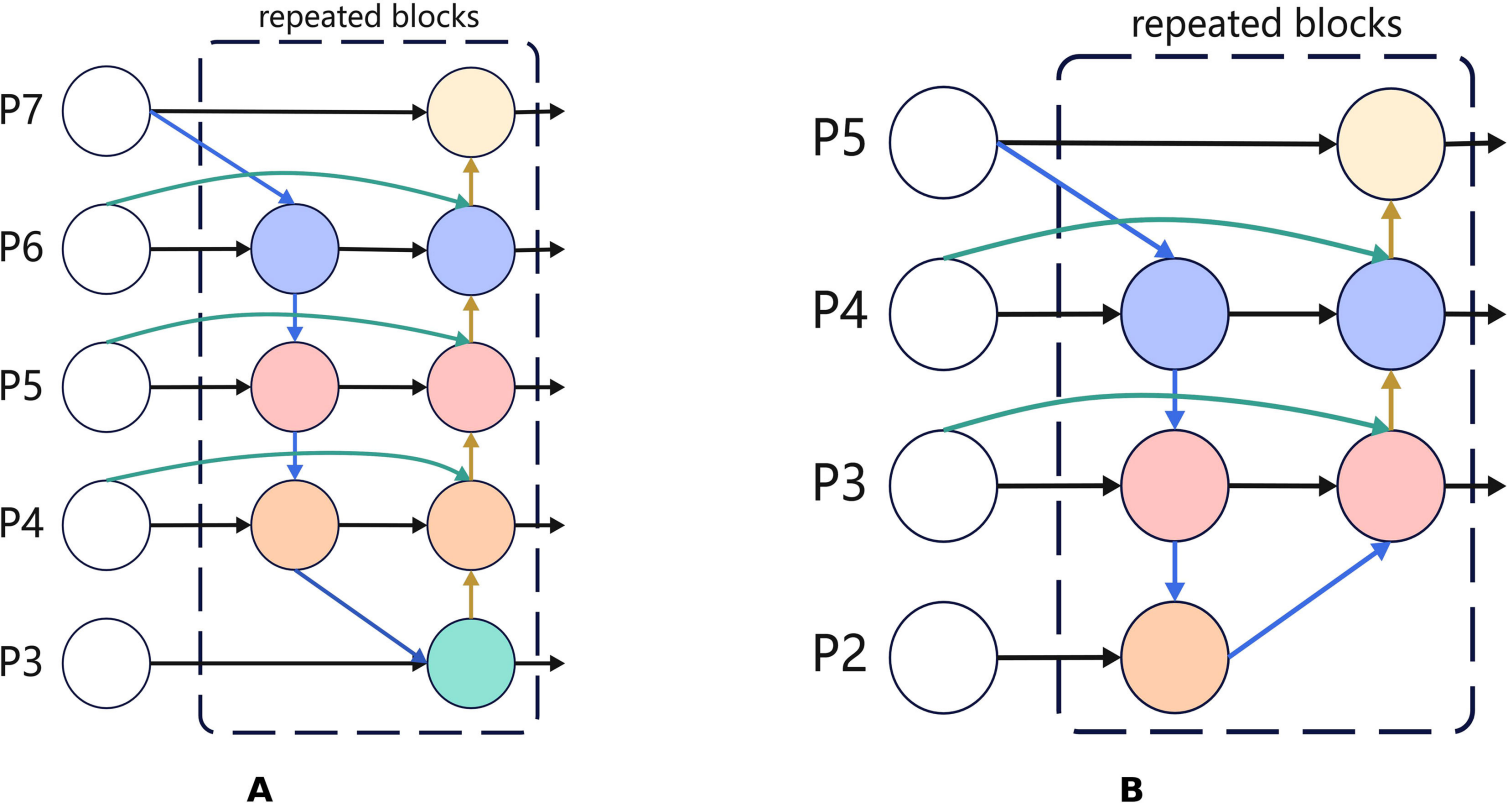
A comparison diagram of the original and improved BiFPN structures. **(A)** shows the original BiFPN based on P3-P7. **(B)** presents the improved version which shifts to P2-P5. It adds learnable weights and depthwise separable convolutions to reduce computation and enhance high-resolution small-object features achieving more balanced bidirectional fusion.

The BiFPN module was modified by translating the original P3- P7 feature hierarchy into a P2 - P5 configuration in order to better accommodate the requirements of smallobject detection. The adapted BiFPN operates on the 256-channel feature maps produced by the ModifiedNeck,i.e. $P_{2}^{\text{out}}∼P_{5}^{\text{out}}$ and employs bidirectional iterative fusion together with a learnable weighting mechanism to efficiently aggregate multiscale features at different scales (Li et al., 2023a). The resulting architecture is depicted in **Figure 5**, and its key features are summarised below:

1. Core computational logic: For the input feature maps *P_i_*,*i* ∈ {2, 3, 4, 5}, learnable scalar weights *w_i_* are applied and normalised to compute a weighted sum. This adaptively amplifies the contribution of the high-resolution P2 features for small object regions and the deep semantic P5 features for complex background regions (Bonte et al., 2023). The core computational formulation is expressed in Equation 4:

(4) $$P_{\text{out}}=\frac{\sum_{i}w_{i}P_{i}}{\sum_{i}w_{i}+Є},\;w_{i}\geq 0$$

Among these, *w_i_* denotes learnable scalar weights constrained to be non-negative via ReLU activation during training, thus avoiding negative contributions to feature fusion (He et al., 2016; Li et al., 2024); *Є* denotes a small constant (set to 1 × 10^−4^ to prevent division by zero and ensure numerical stability); $P_{i}$ represents input feature maps at different spatial resolutions; $P_{\text{out}}$ denotes the fused output feature map.

2. Bidirectional fusion mechanism: The BiFPN implements top-down and bottom-up pathways to propagate features hierarchically. In the top-down pathway, higher-level feature maps are upsampled using nearest-neighbour interpolation and subsequently fused with intermediate and lower-level feature maps via weighted aggregation. In the bottom-up pathway, lower-level features are downsampled using 3×3 max pooling with a stride of 2, and are then subjected to three-way weighted fusion with the corresponding lateral connections and the incoming top-down features. Each fusion node is followed by a depthwise separable convolution and a SiLU activation function (Ramachandran et al., 2017; Howard et al., 2017) to refine the fused representations while maintaining a 64-channel output.

3. Advantages of Hierarchical Adjustment: Compared with the original BiFPN design, which spans P3 - P7, the P2 - P5 configuration offers several advantages. Including the P2 layer preserves higher spatial resolution, enabling the retention of fine-grained details of small targets. The removal of P6 and P7 reduces computational overhead while retaining sufficient semantic richness in the P5 layer. Consequently, the four-tier P2 - P5 feature pyramid strikes a better balance between detection accuracy and computational efficiency, making it more suitable for lightweight, small-object detection in complex orchard environments.

4. Lightweight design: Standard convolutional operations have been replaced by depthwise separable convolutions, which substantially reduces the number of parameters. A shared-weight strategy has also been employed to decrease storage overhead and computational redundancy, thereby improving the model’s suitability for deployment on resource-constrained edge platforms. The BiFPN produces a four-level, 64-channel, enhanced, multi-scale feature pyramid, which is then sent to the detection head for subsequent bounding-box regression and classification.

#### 2.2.3 Head:P2 detection layer

To address the original YOLOv8n model’s limited sensitivity to small objects, a dedicated P2 detection layer was introduced, along with a four-scale detection architecture comprising P2 - P3 - P4 - P5, to ensure precise and stratified coverage of targets of different sizes. To improve detection accuracy for young olive fruits, the design of the detection head was decoupled (Li et al., 2022), whereby each scale-specific output is processed by independent classification and regression branches. Specifically, the feature maps for each detection scale are routed to a classification branch that performs class probability estimation and a regression branch that predicts bounding-box offsets and objectness scores. Both branches employ lightweight convolutional blocks and scale-appropriate anchors to preserve inference efficiency while enhancing localisation and classification performance. The detailed architecture is presented below:

1. Multi-scale detection layer design: The detection head receives four 64-channel feature maps from the BiFPN, with each feature map corresponding to a specific target size range: the P2 layer focuses on ultra-small targets (less than 32×32 pixels), the P3 layer processes small-sized targets (32×32 pixels to 64×64 pixels), the P4 layer is responsible for medium-sized targets (64×64 pixels to 128×128 pixels), and the P5 layer detects large-sized targets (greater than 128×128 pixels). The 160×160 high-resolution feature representation provided by the P2 layer significantly enhances the architecture’s spatial localization accuracy and feature representation capability for small targets. The collaborative design of the P2 detection layer and the decoupled detection head further ensures the efficient utilization of fine-grained details — the regression branch gradually optimizes the localization accuracy of small target bounding boxes through a three-layer convolutional structure, effectively avoiding feature conflicts with the classification task. The classification branch focuses on capturing fine-grained phenotypic features retained by the P2 layer, such as fruit peel texture and local grayscale variations. This enables effective distinction from background vegetation even when the target size is less than 30 pixels.

2. Bounding-box regression branch: An independent regression branch is instantiated for each of the four detection scales to process the 64-channel input feature maps. Each branch uses a three-layer convolutional sequence consisting of a 3 × 3 convolution, a second 3 × 3 convolution and a final 1 × 1 convolution. The first 3×3 layer preserves the 64-channel dimensionality while extracting spatial features, the second 3×3 layer further strengthens the representation of local features, and the final 1×1 layer produces a 64 channel tensor that encodes the prediction features of the bounding box. Progressively refining localisation information through stacked convolutions improves the accuracy of bounding-box regression.

3. Classification prediction branch: This operates in parallel with the regression branch and uses a three-layer convolutional architecture to predict classes. The initial 3 × 3 convolution increases the number of channels from 64 to 80, and the subsequent 3 × 3 convolution further refines the feature representation. A final 1 × 1 convolution then produces an 80-channel tensor of class-confidence scores. Having an independent classification branch reduces feature conflict between the classification and regression tasks, thereby enhancing the overall performance of multi-task learning.

4. Advantages of the decoupled head: Compared with conventional shared-convolution architectures, the decoupled detection head implements separate feature-extraction pathways for classification and regression. This enables the model to learn task-specific representations. The regression branch is optimised for precise bounding-box localisation and therefore emphasises spatial sensitivity, whereas the classification branch is optimised for semantic discrimination and thus emphasises feature separability. By isolating these tasks, mutual interference is avoided, leading to improved detection accuracy and faster convergence during training. This design also allows each branch to be optimised for computational efficiency, which is beneficial for deployment on resource-constrained edge platforms.

5. Loss computation and post-processing: Outputs from the regression branch are decoded into bounding-box coordinates using Distribution Focal Loss (DFL) decoding (Li et al., 2020), whereas outputs from the classification branch are converted into class probabilities via Sigmoid activation. During training, positive and negative samples are assigned using the Task-Aligned Assigner (Feng et al., 2021), which considers classification confidence and IoU quality together to make more reliable sample selections. During inference, predictions from the four detection scales are decoded in an anchor-free manner (Tian et al., 2019), and redundant detections are suppressed using non-maximum suppression (NMS) (Felzenszwalb et al., 2009) to produce the final set of detections. The four-scale detection architecture enables the model to capture targets across multiple spatial resolutions. The inclusion of the high-resolution P2 layer notably improves the Recall and localisation accuracy of small objects.

### 2.3 Evaluation indicators

Accurate detection of olives requires a balance of localisation accuracy, completeness of detection, reliability of results and lightweight deployability; therefore, no single metric can comprehensively characterise model performance. Guided by the standard evaluation criteria used in the object detection community, as well as the specific requirements of olive fruit detection — namely, the presence of a high proportion of small targets and complex backgrounds, and the need for edge deployment — this study adopts seven core metrics: Precision, Recall, the F1 score, mean average precision at 50% (mAP50), parameter count, frames per second (FPS), and giga floating-point operations (GFLOPs). These metrics enable a multifaceted assessment of the proposed YOLO-TinyFuse model across seven dimensions: localisation performance, detection accuracy, overall effectiveness, model complexity, real-time capability and computational efficiency. The corresponding mathematical definitions are given in Equations 5–11:

(5) $$\text{Precision}=\frac{TP}{TP+FP}$$

(6) $$\text{Recall}=\frac{TP}{TP+FN}$$

(7) $$\text{F}1-\text{Score}=2\times \frac{\text{Precision}\times \text{Recall}}{\text{Precision}+\text{Recall}}$$

(8) $$\text{mAP}50=\frac{1}{N}\sum_{i=1}^{N}AP_{i}(\text{IoU}=0.5)\;(N=1,\;\text{Only the category of oil olives})$$

(9) Parameters=out_channels×in_channels×kh×kw+(out_channels if bias=True else 0)

(10) $$\text{FPS}=\frac{N_{\text{total}}}{T_{\text{infer}}}$$

(11) $$\text{GFLOPs}=\frac{\sum_{l=1}^{L}\text{FLOP}s_{l}}{10^{9}}$$

Here, *TP* denotes the number of instances of olive fruit correctly identified by the model. *FP* denotes the number of negative samples, such as branches, specular highlights or other elements of the orchard background, incorrectly classified as olive fruit. *FN* denotes the number of instances of olive fruit incorrectly classified as non-olive; $N_{\text{total}}$ denotes the total number of frames used for FPS evaluation; $T_{\text{infer}}$ denotes the total inference time required by the model to process $N_{\text{total}}$ frames. GFLOPs are calculated by summing the floating-point operations of all layers in the model, where each convolutional layer contributes 2 × C_out × C_in × k_h × k_w × H_out × W_out operations, and dividing by 10^9^. *L* denotes the number of layers in the model. *FLOPs_l_* denotes the floating-point operations of the *l*-th layer.

## 3 Experimental evaluation

### 3.1 Experimental environment

The experimental setup is shown in **Table 1**.

**Table 1:** Experimental environment configuration.

Category	Configuration item	Specific parameters
Hardware Specifications	CPU RAM GPU GPU Memory	Intel (R) Core (TM) i9-13900K (Base Frequency 3.00GHz) 64GB NVIDIA GeForce RTX 4090 24GB
Software Environment	Operating System Programming Language Deep Learning Framework CUDA Version	Linux Python3.8 PyTorch 2.1.0 11.8

### 3.2 Algorithm training

To address the issues of loss of fine-scale features, poor occlusion robustness and interference from complex backgrounds encountered in olive fruit detection, an improved YOLOv8n-based model was developed that integrates the ModifiedNeck, BiFPN and a dedicated P2 high-resolution detection layer. This enhanced model was then trained and compared with the original baseline model. The model’s hyperparameters were selected to optimise scene adaptability and overall performance. Training was conducted for 100 epochs with a batch size of eight and an input resolution of 640 × 640 pixels to balance phenotypic feature retention and computational efficiency (He et al., 2022). The optimiser was configured in auto mode with an initial learning rate of 0.01, a decay coefficient of 0.01, momentum of 0.937 and weight decay of 0.0005 to promote the efficient learning of features of small objects. Data augmentation settings included a mosaic factor of 1.0, a 50% probability of horizontal flipping, and HSV colour space adjustments. Mixup was disabled (mixup = 0.0) to prevent mixing of fruit and background pixels. These settings reduce confusion of small fruit features and effectively increase the relative representation of small target instances, thereby improving the model’s robustness in complex field environments.

### 3.3 Experimental results and analysis

#### 3.3.1 Comparative experiment

The comparative set comprised several representative detection architectures. DETR-R50 uses a ResNet-50 backbone and its four hierarchical feature maps are reduced in channel dimensionality using 1 × 1 convolutions. These are then concatenated to form a single-scale feature map with a stride of 32, which is then input to the Transformer encoder. DETR-R50-DC5 (Carion et al., 2020) is also based on ResNet-50, but it uses a DC5 modification which removes conv5 x downsampling in order to preserve a single-scale feature map with a stride of 16. YOLOv5n (Jocher et al., 2020) uses a CSPDarknet backbone, combining CSP modules with an FPN+PAN neck to balance computational cost while achieving multi-scale feature fusion. YOLOv9t (Wang et al., 2024) further refines feature propagation on an enhanced CSPDarknet backbone by adjusting the width of the channels and the size of the convolutional kernels to balance inference speed and representational capacity. YOLOv11n (Jocher et al., 2024) uses a modified CSPDarknet backbone with simplified structures to reduce computational overhead. It also uses an optimised FPN+PAN and detection-head hierarchy to improve detection of small objects. Comparative results are presented in **Table 2**, which reports Precision, Recall, GFLOPs and mAP50 for each model, and visually demonstrates the relative detection advantages of YOLO-TinyFuse.

**Table 2:** Comparison of different object detection models.

Model	Precision(%)↑	Recall(%)↑	GFLOPs↓	mAP50(%)↑
Yolov5n	85.3	81.0	7.18	89.4
Yolov9t	84.8	80.4	8.48	88.7
Yolov11n	85.7	81.3	**6.44**	89.7
DetrR50	34.4	79.3	36.82	68.5
DetrR50-Dc5	33.4	78.2	62.32	66.3
YOLO-TinyFuse	**86.9**	**84.5**	20.41	**92.3**

The bold value means the highest value, the symbol (↑) means the higher value is better, and the symbol (↓) means the lower value is better.

Comparative experiments demonstrate that YOLO-TinyFuse achieves superior performance to DETR-R50-DC5, DETR-R50, YOLOv5n, YOLOv9t and YOLOv11n in terms of Precision, Recall, GFLOPs and mAP50 metrics. YOLO-TinyFuse achieves an mAP50 of 0.923, indicating substantially higher detection accuracy at an intersection over union (IoU) of 0.5 than the evaluated baselines. The model achieves a GFLOPs of 20.41, indicating substantially lower computational cost than the evaluated baselines. The model’s Recall is 0.845, reflecting a significant reduction in missed detections of true targets. Meanwhile, its Precision is 0.869, indicating a low false-positive rate, thereby improving the validity and reliability of the detection outputs.

**Figure 6:**
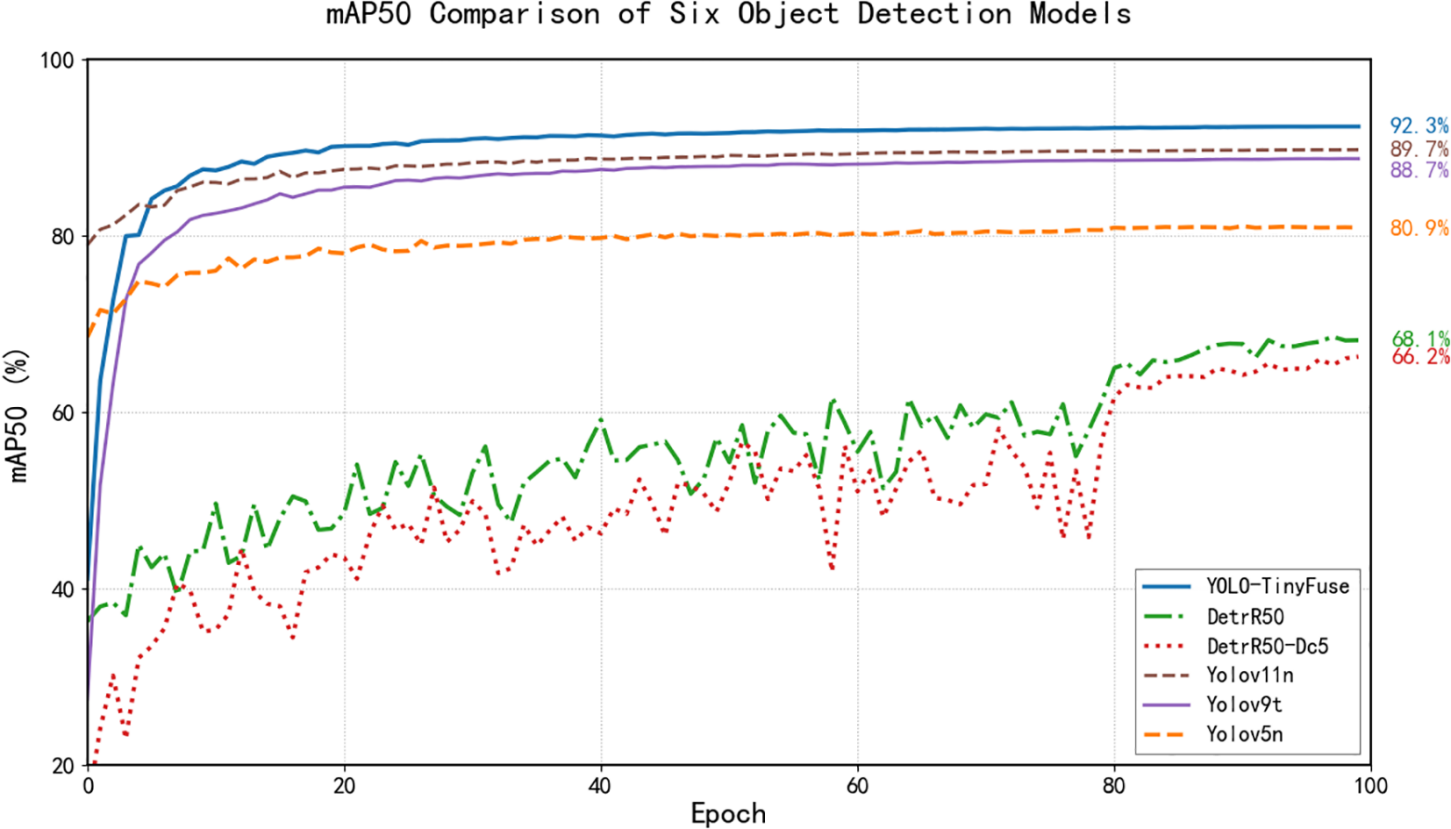
mAP50 comparison of six object detection models. Performance on the olive test set shows YOLO-TinyFuse outperforms DETR-R50 DETR-R50-Dc5 YOLOv5n YOLOv9t and YOLOv11n. This validates the effectiveness of the proposed modules.

Convergence analysis based on comparative experiments indicates that YOLO-TinyFuse achieves an mAP50 of 0.923. This corresponds to increases of 26.0, 23.8, 2.9, 3.6 and 2.6 percentage points relative to DETR-R50-DC5, DETR-R50, YOLOv5n, YOLOv9t and YOLOv11n respectively. As shown in **Figure 6**, the mAP50 trajectories recorded during training emphasise YOLO-TinyFuse’s superiority in accurate recognition and localisation, and demonstrate a marked improvement in overall detection performance.

#### 3.3.2 Comparison of YOLO-TinyFuse with the Yolov8n baseline model

**Figure 7:**
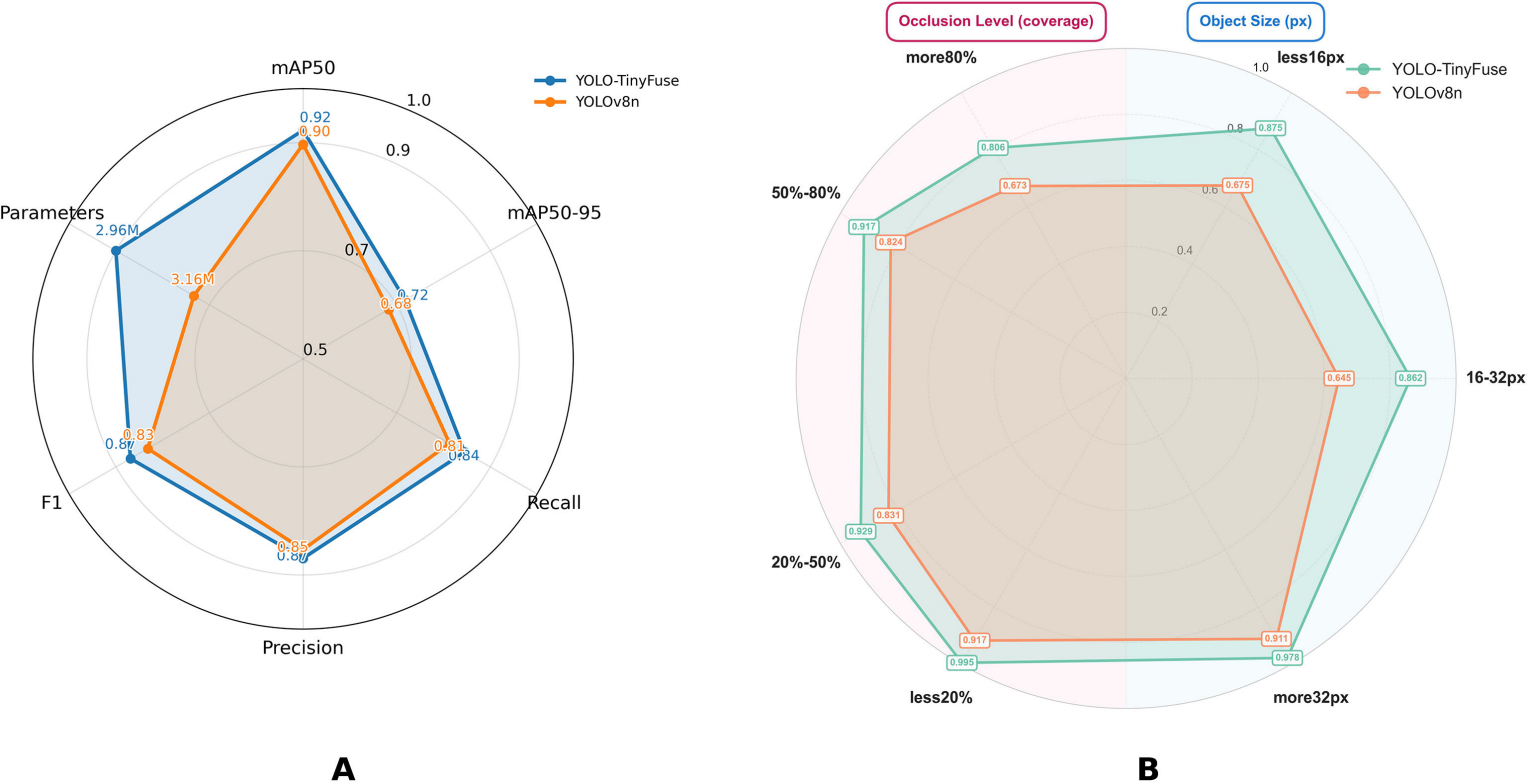
Performance comparison of YOLO-TinyFuse and YOLOv8n. **(A)** Overall performance metrics. **(B)** Performance by occlusion level and object size. YOLO-TinyFuse improves mAP50 by 2.6%, mAP50–95 by 3.5, and F1 by 3.7% compared to YOLOv8n, while reducing parameters by 6.3% demonstrating a balance of accuracy and compactness. The analysis of mAP50 across target sizes (*<*16 px, 16–32 px, *>*32 px) demonstrates that the P2 layer effectively addresses small object detection challenges. Furthermore, YOLO-TinyFuse achieves higher mAP50 values across all occlusion levels (*<*20%, 20–50%, 50–80%, *>*80%), which confirms that the BiFPN module enhances detection robustness under varying occlusion conditions.

The detection performance of the different model variants was evaluated using core metrics on the test set. **Figure 7** shows how YOLOv8n and YOLO-TinyFuse compare across key indicators, including mAP50, mAP50-95, F1 score and parameter count. YOLO-TinyFuse shows improvements of 2.6% in mAP50, 3.5% in mAP50–95 and 3.7% in the F1 score compared with YOLOv8n, while reducing the number of parameters by 6.33%. These results suggest that YOLO-TinyFuse offers superior detection performance and greater model compactness than YOLOv8n.

In addition, **Figure 7** further analyzes performance by object size (less than 16 px, 16–32 px, more than 32 px) and occlusion level (less than 20%, 20-50%, 50-80%, more than 80%). The results indicate that YOLO-TinyFuse consistently outperforms YOLOv8n across all object size categories, which demonstrates that the P2 layer effectively mitigates the challenges of small object detection. Meanwhile, YOLO-TinyFuse achieves higher mAP50 values across all occlusion levels, which confirms that the BiFPN module enhances detection robustness under varying occlusion conditions.

**Figure 8:**
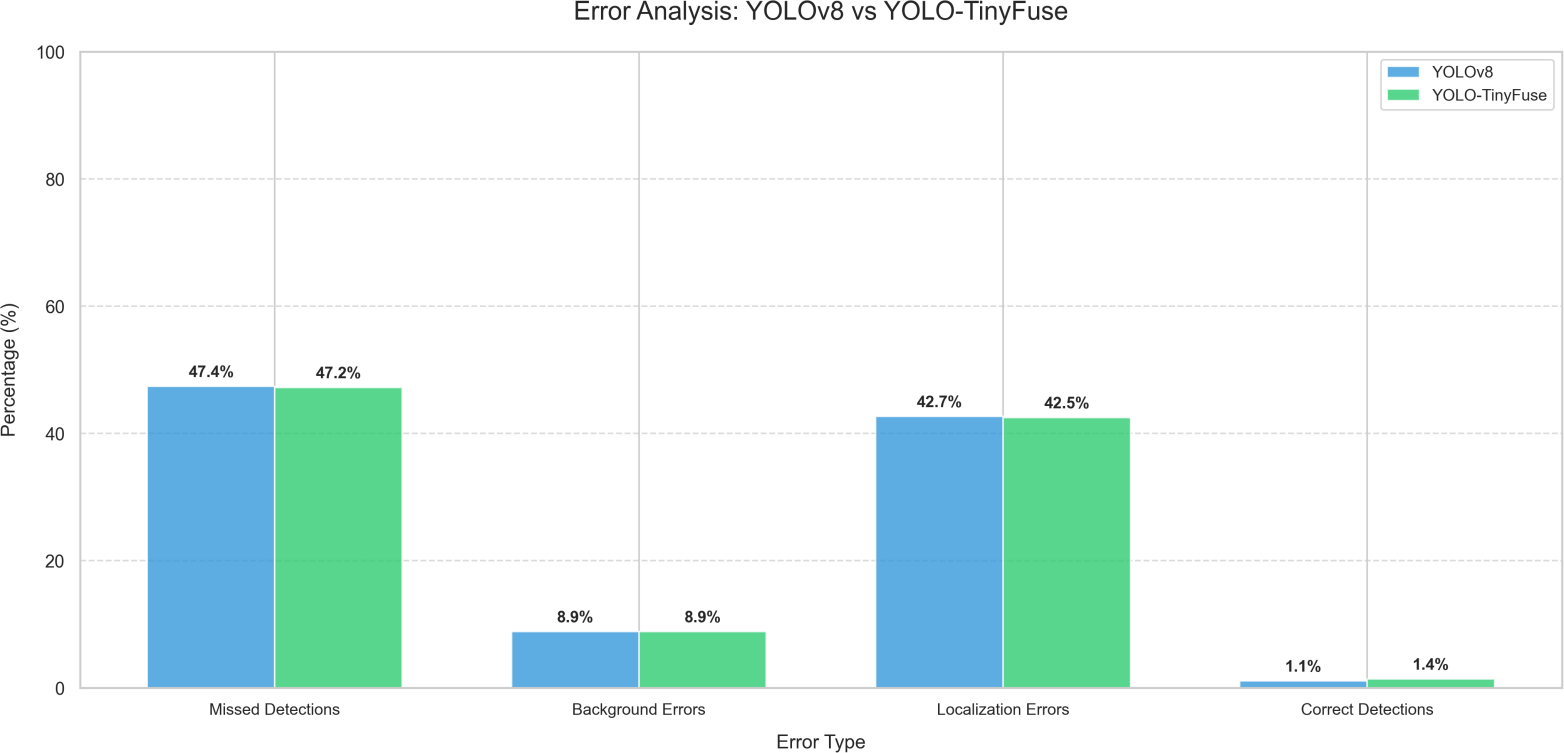
COCO-style error analysis comparison: YOLOv8n vs YOLO-TinyFuse. The error analysis categorizes detection failures into four types following COCO evaluation methodology: Missed detections, Background errors, Localization errors, Correct detections.

To provide deeper insights into detection failures and validate the effectiveness of the P2 + BiFPN combination in addressing small object and occlusion challenges, a comprehensive COCO-style error analysis was conducted following the methodology proposed. As shown in **Figure 8**, the error analysis categorizes detection failures into four types: missed detections, background errors, localization errors, and correct detections. Compared with YOLOv8n, YOLO-TinyFuse achieves optimization in detection error metrics: missed detections reduced from 47.4% to 47.2%, localization errors reduced from 42.7% to 42.5%, and correct detections increased from 1.1% to 1.4%. Although the improvements are modest, the systematic reduction in error rates, particularly in missed detections and localization errors, validates that the P2 + BiFPN combination effectively mitigates small object detection challenges and occlusion problems by enhancing feature representation and cross-scale fusion.

#### 3.3.3 Melting experiment

The experiment was designed to quantify the performance contributions of three modifications: including a P2 high-resolution layer to improve the representation of small objects, incorporating a ModifiedNeck to optimise cross-scale feature fusion and integrating a BiFPN module to strengthen multi-scale feature interaction. A set of ablation experiments was conducted using YOLOv8n as the baseline to evaluate the independent effects and synergistic impact of these components. Each model variant was evaluated using the validation set, and the influence of each module on Precision, Recall, mAP50 and mAP50–95 was measured. The quantitative results are presented in **Table 3**.

**Table 3:** Ablation study results.

BiFPN	ModifiedNeck	P2	Precision(%)↑	Recall(%)↑	mAP50(%)↑	mAP50-95(%)↑
			85.3	81.3	89.7	68.3
✓			85.5	83.1	91.1	68.7
	✓		85.2	82.4	90.6	68.0
		✓	85.5	82.9	91.0	69.0
	✓	✓	85.9	84.3	92.0	71.4
✓		✓	86.7	84.3	92.2	71.3
✓	✓		85.2	82.8	91.0	68.6
✓	✓	✓	**86.9**	**84.5**	**92.3**	**71.8**

✓represents the use of this module in the model. The bold value means the highest value and the symbol (↑) means the higher value is better.

Ablation experiments indicate that the YOLOv8n baseline achieved Precision of 0.853, Recall of 0.813, mAP50 of 0.897 and mAP50–95 of 0.683. The isolated addition of BiFPN produced modest improvements across all metrics, with Recall increasing by 1.8 percentage points. Notably, the isolated addition of ModifiedNeck leads to a 0.3 percentage point decrease in mAP50-95, which is mainly caused by two key factors. First, the ModifiedNeck is designed to standardize feature channel dimensions via 1×1 convolutions and optimize cross-scale alignment through bidirectional propagation, but without the high-resolution spatial details provided by the P2 layer, the bottom-up feedback pathway of ModifiedNeck can only transmit low-quality downsampled features, where fine-grained cues such as edge and texture information of small olive fruits are severely blurred. Second, the bidirectional feature flow of ModifiedNeck introduces additional computational steps without complementary spatial information, which not only increases model complexity but also amplifies noise interference in mid-level semantic features, thereby reducing the detection confidence of low-to-medium confidence targets. In contrast, incorporating the P2 layer alone resulted in significant improvements, with mAP50–95 rising by 0.7 percentage points. These observations suggest that augmenting a single module offers limited benefit and reveals inherent limitations in olive-fruit recognition.

For two-module combinations, the ModifiedNeck plus P2 configuration delivered increases of 1.0, 2.4 and 1.4 percentage points in mAP50, mAP50–95 and Recall respectively, thereby validating the effectiveness of bidirectional feature propagation. However, a significant negative synergy effect is observed in the combination of BiFPN and ModifiedNeck. Without the P2 layer, although the metrics of this combination are slightly improved compared to using ModifiedNeck or the P2 layer alone, they are all inferior to those of BiFPN used independently. The core reason for this phenomenon lies in the inherent differences in core propagation mechanisms and functional positioning between the two modules, despite both focusing on cross-scale feature fusion: BiFPN centers on dynamic weighted fusion, enhancing the contribution of high-level semantic features through learnable weights and achieving lightweight design via depthwise separable convolutions; ModifiedNeck focuses on channel unification and bidirectional path connectivity, standardizing feature dimensions through 1×1 convolutions and optimizing cross-scale alignment of low-level spatial features via bidirectional flows. In the absence of high-resolution spatial cues provided by the P2 layer, the two modules suffer from functional mismatch due to the lack of feature complementarity—the detailed information fed back by ModifiedNeck from bottom to top is already insufficient, and BiFPN’s weighting mechanism further suppresses mid-level spatial features, leading to a disconnect between fine-grained details and semantic context fusion. Coupled with redundant computations and noise interference caused by repeated bidirectional processing, the detection accuracy of low-to-medium confidence targets is ultimately reduced, resulting in all metrics of the combined model being lower than those of the single BiFPN module.

The three-module integrated system (BiFPN + ModifiedNeck + P2) achieved the best overall performance: Precision is 0.869, Recall is 0.845, mAP50 is 0.923, and mAP50–95 is 0.718. Compared with the ModifiedNeck-only variant, the three-module model improved mAP50–95 by 3.8 percentage points and Recall by 2.1 percentage points. These results collectively confirm that single-module enhancements are limited, that module combinations provide substantial synergistic gains when paired with the P2 layer to mitigate functional conflicts, and that three-module integration yields optimal performance. This offers a clear direction for optimising small-object detection in agricultural phenotyping.

#### 3.3.4 Visualization analysis

**Figure 9:**
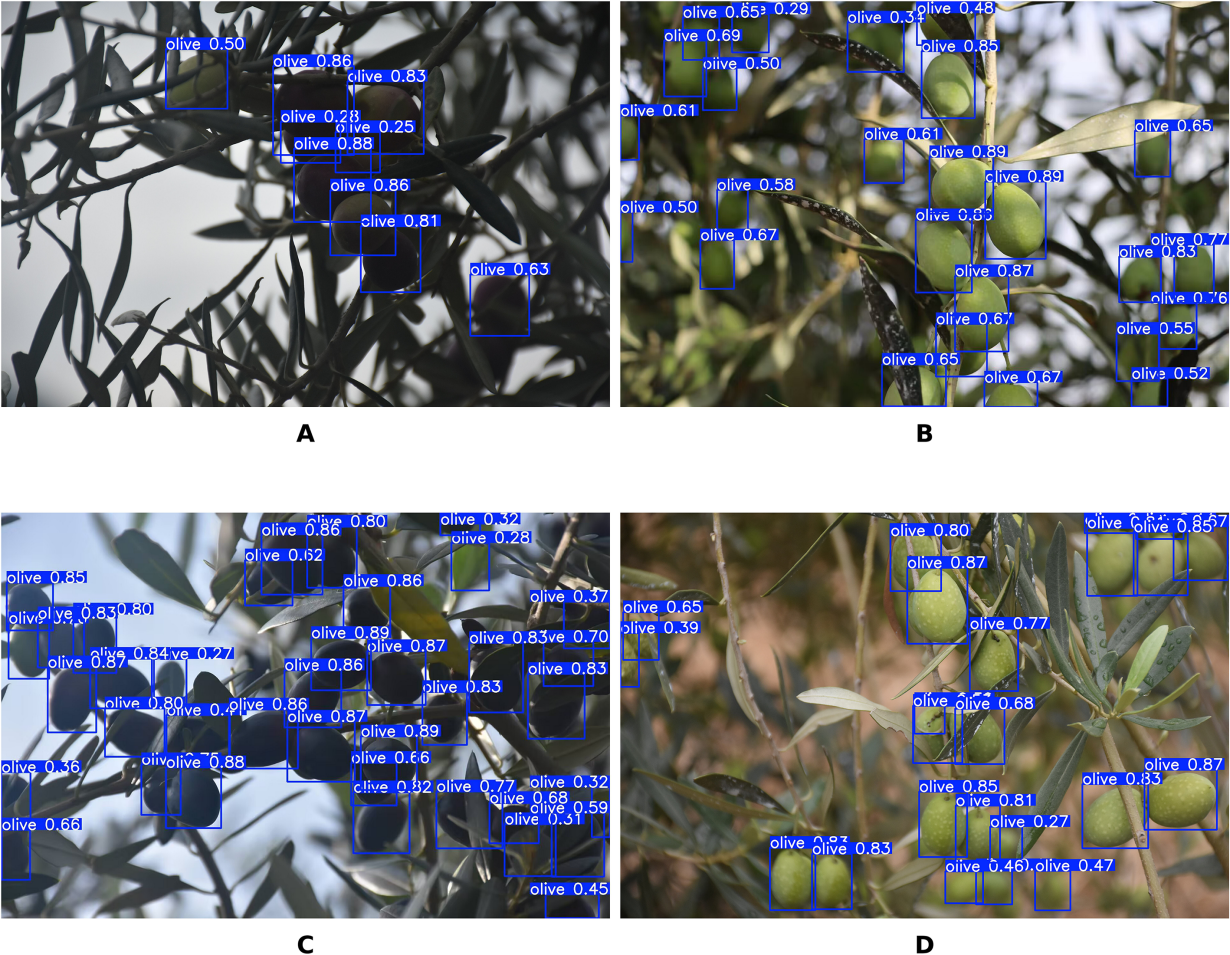
Target detection results in different environments. **(A)** Low-light Environment. **(B)** High-brightness Environment. **(C)** Fruit Obstruction. **(D)** Fruit Blur.

Distributional inconsistencies arising from domain shifts caused by illumination variation and atypical target appearance can degrade detection performance. Representative detection examples under diverse environmental conditions (**Figure 9**) demonstrate the YOLO-TinyFuse model’s robustness across multiple scenarios. Even in challenging circumstances, such as low illumination and blurring caused by occlusion, the model maintains relatively high confidence scores for true targets, demonstrating its strong adaptability and resilience to common field perturbations.

**Figure 10:**
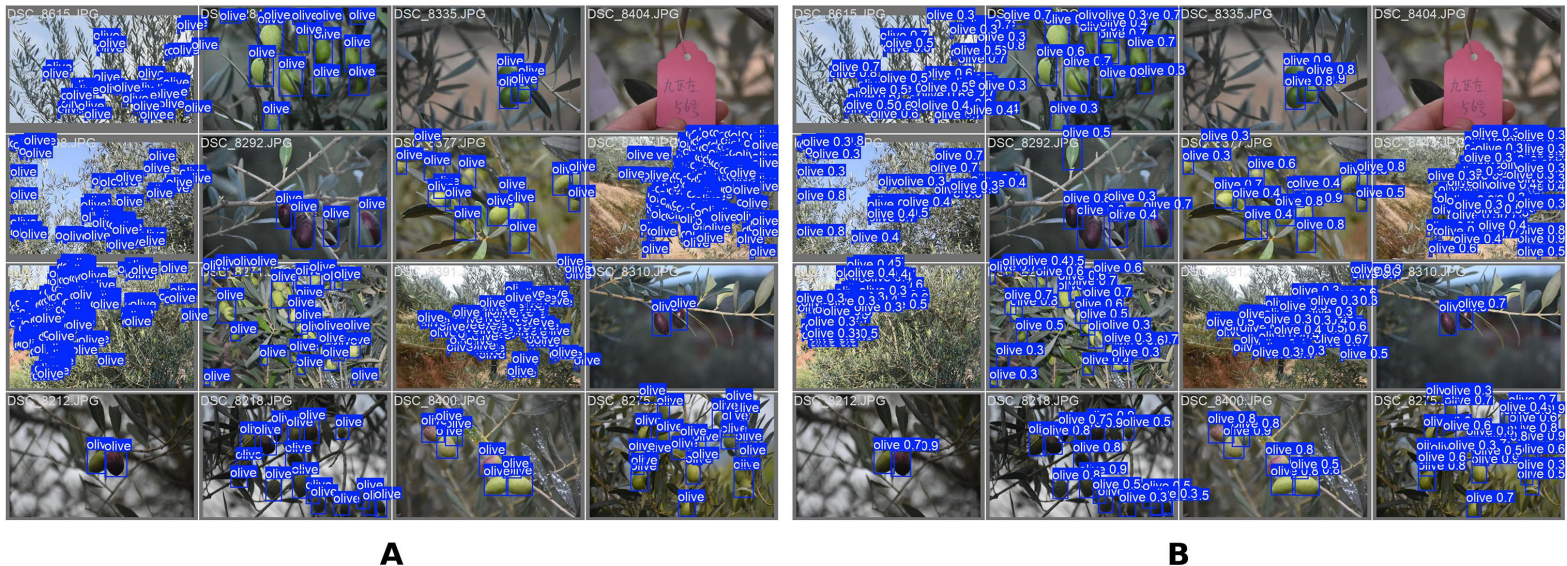
Comparison chart of manual annotation and model prediction results. **(A)** shows human-annotated ground truth. **(B)** presents model predictions. The side-by-side view highlights alignment on multi-scale olive fruits and differences in misses and false alarms enabling intuitive accuracy assessment.

The comparison between manual annotations and model predictions (**Figure 10**) provides quantitative validation of the detection performance. In the figure, val batch0 labels denotes the ground-truth annotations produced by human annotators. These encompass olive fruits of varying sizes and morphologies and serve as the evaluation benchmark.val batch0 pred denotes the model’s predicted detections and reflects its capacity for phenotypic feature representation and object recognition. Direct comparison of these two sets of annotations allows prediction accuracy to be assessed intuitively and further substantiates the model’s learning effectiveness and generalisation capability under diverse field conditions.

**Figure 11:**
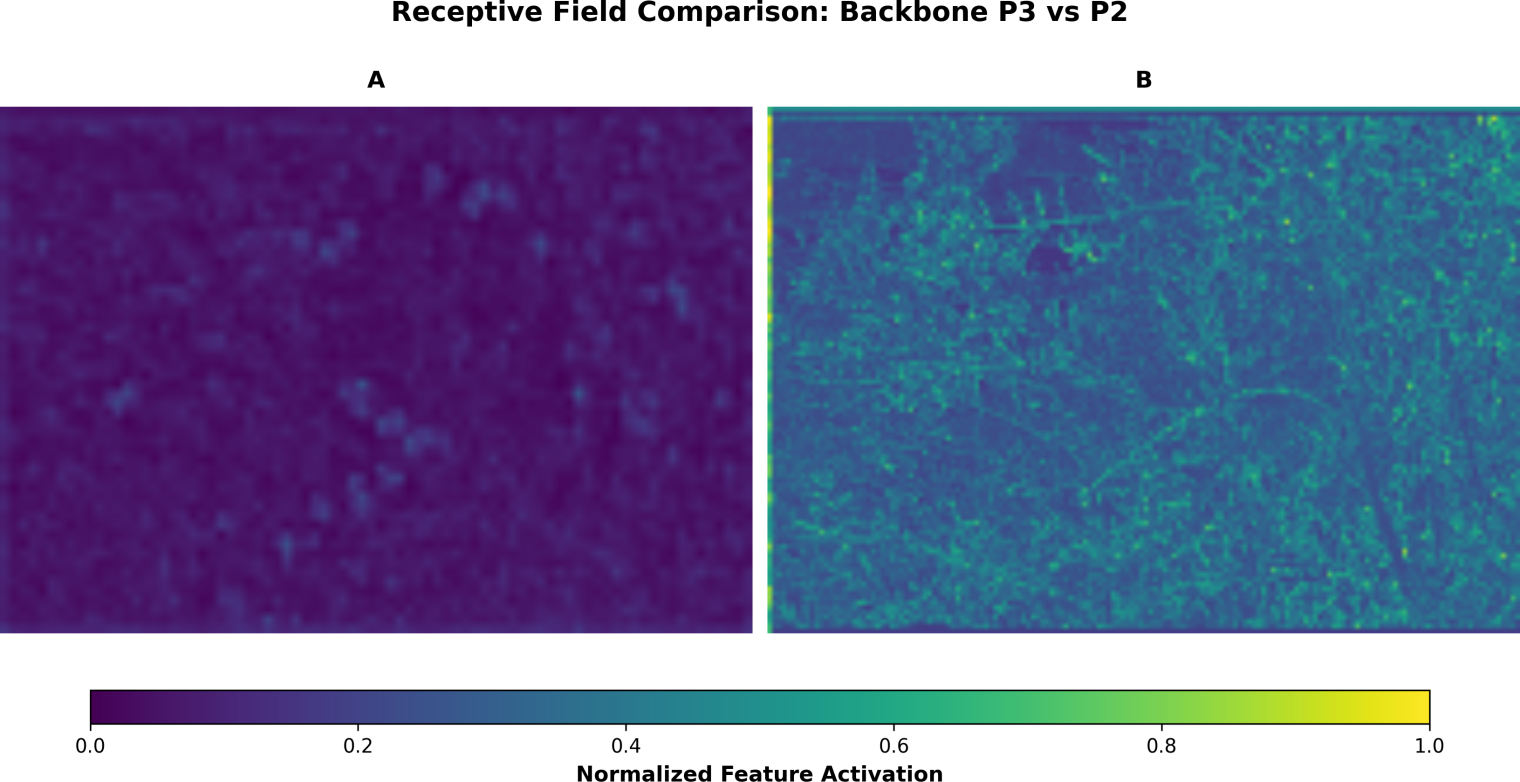
Contrast Map of the Receptive Field. **(A)** YOLOv8n at P3 feature layer; **(B)** YOLO-TinyFuse at P2 feature layer. Comparing YOLOv8n at P3 with YOLO-TinyFuse at P2 reveals more focused and stronger activations at P2. This indicates the high-resolution branch better captures small-object details.

To complement the visualisation analysis, **Figure 11** shows the differences in receptive field distribution between YOLO-TinyFuse and YOLOv8n at the P3 and P2 feature levels. Panels (a) and (b) show the feature activation maps for the backbone of YOLOv8n at the P3 level (80 × 80) and YOLO-TinyFuse at the P2 level (160 × 160), respectively. These visualisations show that activations at the P2 level in YOLO-TinyFuse are more spatially concentrated and have a stronger response, proving that despite the model being compressed to 2.96 million parameters, the modified backbone can still capture the fine-grained phenotypic features of small olive fruits, thereby improving the accuracy of small object detection.

**Figure 12:**
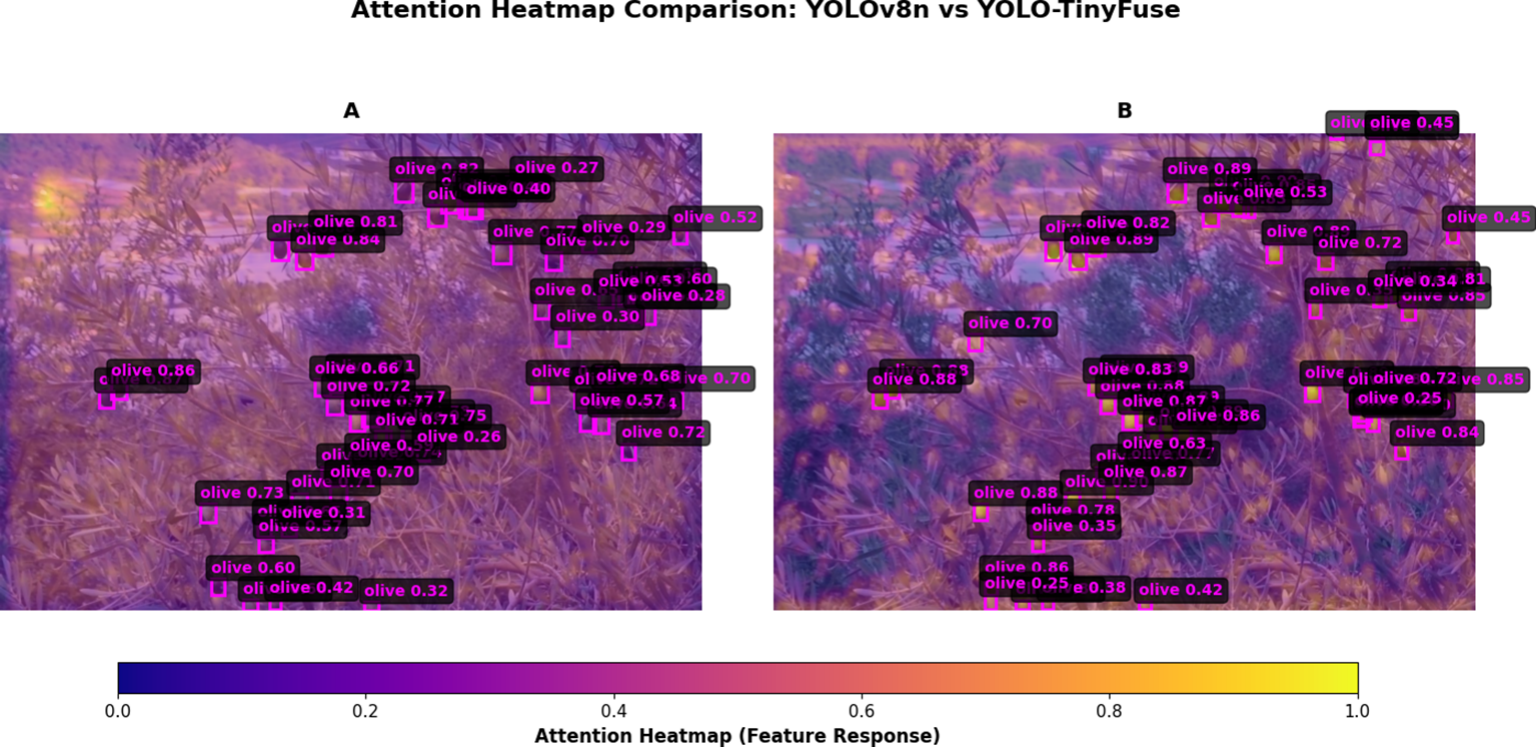
Heatmap Comparison Chart. **(A)** YOLOv8n. **(B)** YOLO-TinyFuse. Heatmaps show YOLO-TinyFuse concentrates high-intensity responses on olive regions while suppressing background. This demonstrates that bidirectional weighted fusion and lightweight design enhance saliency and confidence.

The comparative heatmap analysis (**Figure 12**) further illustrates the efficiency of the lightweight architecture. The visualisations show that YOLO-TinyFuse produces more concentrated, higher-intensity feature responses within the olive-fruit regions. The heatmap is predominantly shifted towards higher activation values, which correspond to increased object-confidence scores. These observations suggest that the model’s lightweight design effectively focuses representational capacity on salient regions while suppressing background interference. The model achieved an inference speed of 18.57 frames per second when deployed on a Raspberry Pi 4B (4GB RAM version) at the target edge. The experimental environment was configured with Raspbian operating system, PyTorch Mobile framework, and OpenCV-based hardware acceleration. Average value calculated from 1000 consecutive frames, with a standard deviation of ±0.3 frames per second. Corroborates the accuracy of the model’s target localization and the high confidence of its output results, thereby enhancing the detection accuracy and reliability in practical hardware deployment.

In summary, the comprehensive visualisation framework, which encompasses quantitative detection performance, direct comparison between manual annotations and model predictions, receptive-field analysis and attention/heatmap inspection, provides convergent evidence that YOLO-TinyFuse exhibits strong adaptability, high accuracy and computational efficiency for olive fruit detection. The visual analyses corroborate the model’s superior detection accuracy and highlight its suitability for use with hardware that has limited resources, thanks to concentrated feature responses and high-confidence outputs. This supports the practical deployment of the model in agricultural production systems.

## 4 Discussion

### 4.1 Key technical contributions of YOLO-TinyFuse

The enhanced YOLO-TinyFuse model proposed in this study maximizes parameter simplicity while simultaneously improving recognition accuracy and reducing false negative rates. By integrating a P2 fine-grained detection layer, a Bidirectional Feature Pyramid Network (BiFPN) feature fusion mechanism, a ModifiedNeck cross-scale fusion strategy, and a decoupled detection head, the model’s capability to detect small and partially occluded targets is significantly enhanced. The P2 layer extends the detection range to the shallow feature map, which retains richer texture details and spatial location information of small olive fruits—information that is easily lost in deep feature downsampling. This mechanism ensures that the model captures fine-grained features of small targets at the early stage of feature extraction, laying a foundation for accurate localization. Furthermore, its lightweight architectural design reduces computational costs while improving detection efficiency, thus meeting the requirements of practical agricultural applications. Experimental results demonstrate that YOLO-TinyFuse outperforms YOLOv8n across multiple key performance metrics, validating the effectiveness of the proposed improvements. Benefiting from the lightweight design and reduced parameter count, the model achieves synchronous improvements in accuracy and computational efficiency. Specifically, the integration of the P2 layer substantially enhances small target detection performance, while the ModifiedNeck module optimizes cross-scale feature fusion within the Feature Pyramid Network (FPN) structure, strengthening feature transmission and reducing interference from non-informative representations. The ModifiedNeck introduces cross-scale attention mechanisms to adaptively weight feature maps of different scales, prioritizing the fusion of discriminative features related to olive fruits and suppressing redundant background information. This adaptive weighting mechanism solves the problem of uneven feature contribution in traditional FPN, where shallow and deep features are fused with equal weight regardless of their informativeness. In addition, the BiFPN module significantly improves feature utilization efficiency and amplifies the contribution of salient features through bidirectional feature propagation. Unlike unidirectional FPN, BiFPN establishes bidirectional connections between adjacent feature layers, enabling top-down semantic feature propagation and bottom-up detail feature feedback. This bidirectional interaction enriches the semantic information of shallow detail features and supplements the spatial detail information of deep semantic features, forming a more comprehensive feature representation for occluded olive targets.

Beyond the aforementioned modules, the adoption of a decoupled detection head constitutes another critical technical contribution. Compared with conventional shared convolution architectures, the decoupled detection head designs independent feature extraction pathways for classification and regression tasks, enabling the model to learn task-specific representations. The regression branch is optimized for accurate bounding box localization, thereby emphasizing spatial sensitivity, while the classification branch is optimized for semantic discrimination, thus focusing on feature separability. By isolating these two tasks, mutual interference is avoided, leading to improved detection accuracy and accelerated training convergence. The fundamental mechanism behind this improvement lies in the distinct feature requirements of classification and regression: classification relies on global semantic features to distinguish olive fruits from backgrounds and other objects, while regression depends on local spatial features to precisely locate the bounding box. The shared convolution in traditional heads forces a single feature map to satisfy both requirements, resulting in a trade-off between semantic discrimination and spatial localization. The decoupled design eliminates this trade-off by allocating dedicated pathways, allowing each branch to converge to its optimal feature space. This design also allows independent optimization of each branch for computational efficiency, facilitating deployment on resource-constrained edge devices—a core requirement for on-site olive orchard detection.

Ablation experiments further verify the effectiveness of the multi-module collaborative optimization strategy, including the synergistic effect between the decoupled detection head and other modules. The combination of ModifiedNeck and BiFPN mitigates the “semantic-detail disconnection” commonly existing in traditional FPN architectures. This disconnection arises because traditional FPN only conducts one-way feature transmission, leading to the loss of detail information in deep semantic layers and insufficient semantic information in shallow detail layers. The ModifiedNeck’s adaptive weighting and BiFPN’s bidirectional propagation work synergistically to bridge this gap: BiFPN provides bidirectional feature flow, and ModifiedNeck optimizes the fusion weight of each feature component, ensuring that semantic and detail information are fully integrated across scales. The synergistic effect of these two modules, coupled with the performance enhancement of the decoupled detection head, increases the mean Average Precision at 50% intersection over union (mAP50) for small target detection by 1.3 percentage points. Tailored to the small size of olive fruits, the P2 layer alleviates the underfitting problem caused by limited small target samples and significantly improves the Recall of small targets. This is because the P2 layer extracts features from the shallowest feature map with the highest resolution, capturing more pixel-level details of small olive fruits. For small target samples with limited quantity, these detailed features provide more discriminative information, reducing the model’s reliance on large sample sizes and thus alleviating underfitting. In addition to performance improvements, the model achieves a substantial reduction in size and reduces reliance on high-performance computing hardware, meeting the real-time detection requirements of olive orchards. The lightweight design also lowers training costs and inference time, reduces the risk of overfitting, and enhances detection accuracy and stability. Collectively, these improvements—including the decoupled detection head, P2 layer, BiFPN, and ModifiedNeck—demonstrate that the model strikes a balance between high performance and computational efficiency.

**Figure 13:**
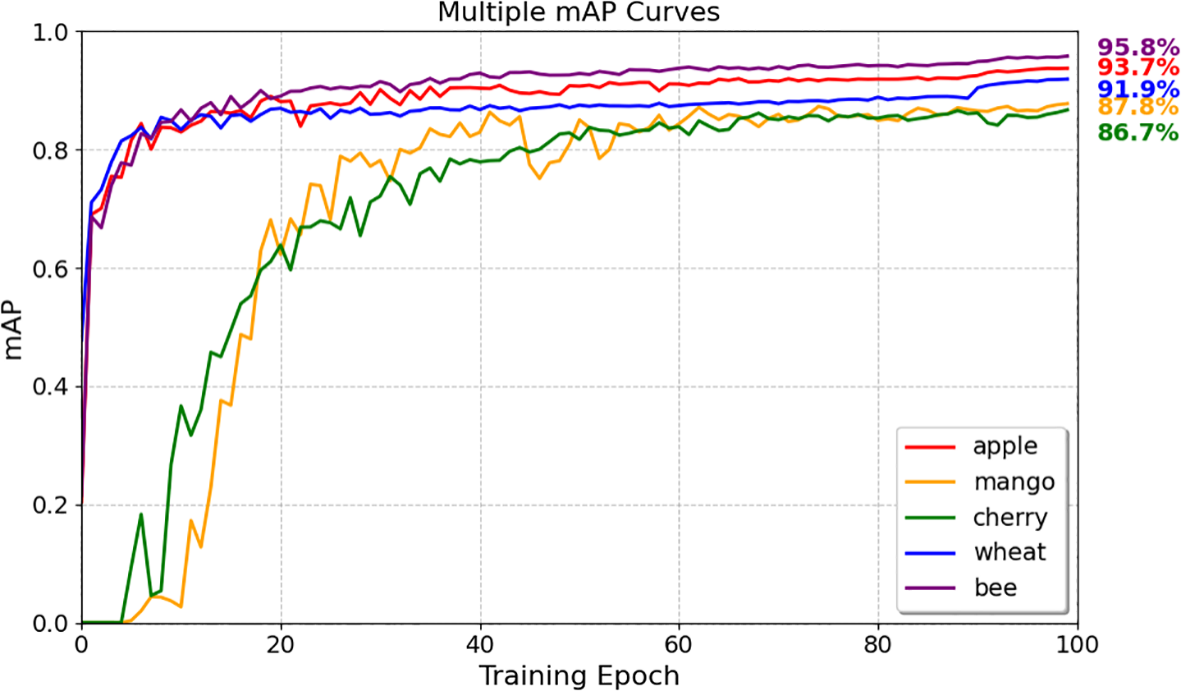
Changes in mAP50 for different targets during training. Training curves on apple mango cherry wheat and bee show the single architecture converges quickly. It achieves high mAP50 across diverse small and occluded targets demonstrating strong reusability.

The YOLO-TinyFuse model demonstrates strong adaptability in a variety of scenarios, enabling efficient detection of olives while generalising effectively to a wide range of agricultural and biological tasks involving small objects, including wheat, cherries, bees, mangoes and apples. The datasets for these small-target and high-occlusion crops were obtained from Kaggle, with specific dataset sources referenced as follows: wheat dataset (enddl22, 2024), cherry dataset (enddl22, 2024), bee dataset (Andrew, 2024), mango dataset (enddl22, 2024), and apple dataset (Projects, 2024). For each target, the model was trained from scratch on their respective datasets with consistent experimental settings: 100 training epochs, a batch size of 8, and identical hyperparameters including learning rate, weight decay, and momentum. Specifically, wheat, cherries, bees, mangoes, and apples were all subjected to independent training from scratch with the aforementioned uniform configuration. As shown in **Figure 13**, the model achieves highly precise recognition of dense small targets, with mAP50 values of 91.9% for wheat, 86.7% for cherries, and 95.8% for bees. This meets the demands of real-time detection in field environments characterised by high object density. For crops with substantial occlusion, such as mangoes and apples, the model achieves stable detection performance, with mAP50 values of 87.76% and 93.74% respectively.

**Figure 14:**
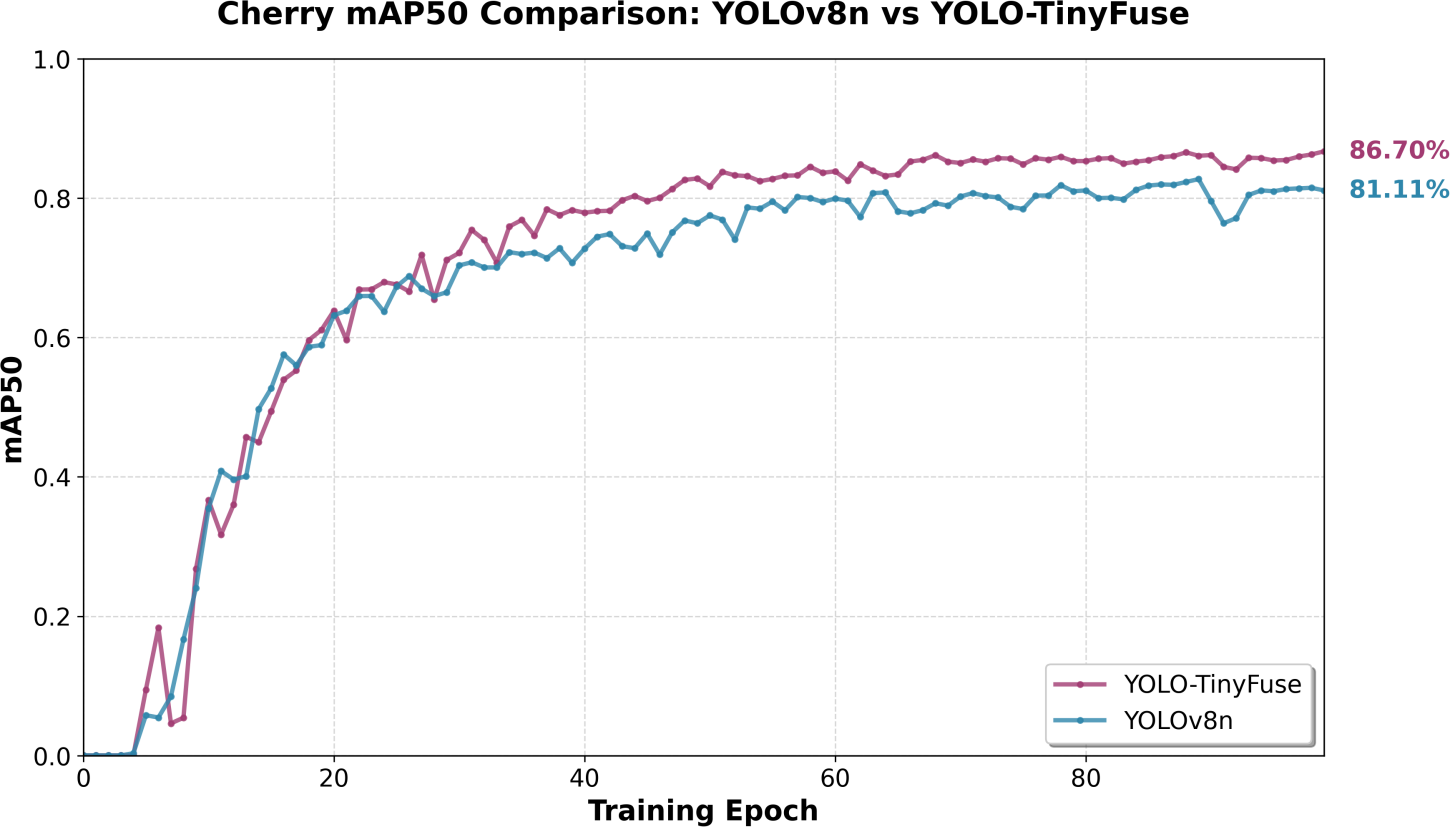
Cherry mAP50 comparison: YOLOv8n vs YOLO-TinyFuse. YOLO-TinyFuse achieves higher mAP50 than YOLOv8n, indicating improved performance for cherry detection.

**Figure 15:**
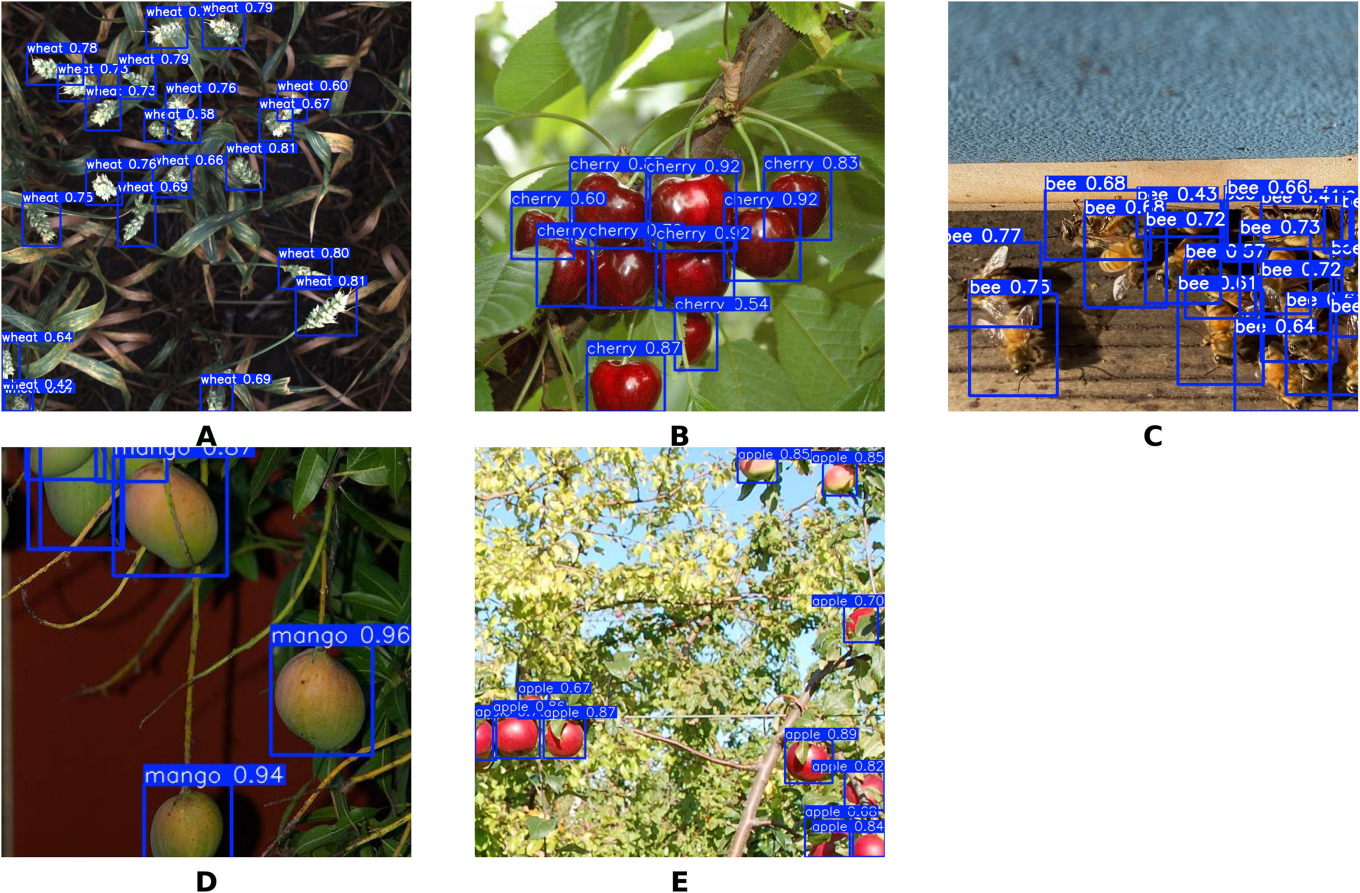
Visual detection results of YOLO-TinyFuse on multiple agricultural and biological targets. **(A)** Wheat. **(B)** Cherry. **(C)** Bee. **(D)** Mango. **(E)** Apple.

To provide a detailed comparative analysis demonstrating the model’s superiority over the baseline, cherry detection was selected as a representative case study. Cherries were chosen for this comparison because they exhibit typical characteristics of small agricultural targets—including high density, variable occlusion levels, and morphological similarity to background elements—while also presenting moderate complexity that allows clear visualization of performance differences between YOLO-TinyFuse and YOLOv8n. As illustrated in **Figure 14**, YOLO-TinyFuse demonstrates superior detection performance compared to YOLOv8n, with improved precision in identifying small cherry targets and reduced false positives in complex orchard backgrounds. Visual examples of detection results across all evaluated crops, including wheat, cherries, bees, mangoes, and apples, are presented in **Figure 15**, showcasing the model’s consistent performance across diverse agricultural scenarios.

With only 2.96 million parameters, the model can be rapidly adapted to different crop detection tasks without any structural modifications, overcoming the traditional limitation of single-crop-specific models. These results provide an efficient, reusable technical foundation for developing unified, multi-crop detection systems for specialised agricultural production.

### 4.2 Structural comparison with recent agricultural small object detectors

To further highlight the advantages of YOLO-TinyFuse in structural design and practical applicability, this section conducts a systematic comparison with three representative recent agricultural small-object detectors, namely YOLOv8-p2, Slim-BiFPN, and Lightweight Transformer. The comparison focuses on core structural designs, technical focuses, performance trade-offs, and scenario adaptability, without being limited to numerical differences in metrics. The key comparative analysis is as follows:

1. Structural Design and Technical Focus Comparison

YOLOv8-p2 is based on the YOLOv8n backbone, adopts MobileNetV3 for lightweight optimization, integrates BiFPN for feature fusion, and adds a P2 high-resolution detection head to enhance small-target detail capture (Xu et al., 2024). Its core technical focus is “shallow feature retention + multi-scale fusion”, but it lacks targeted optimization for cross-scale feature weight adaptation and task decoupling. The EMA attention mechanism embedded in the C2f module mainly enhances local feature extraction, without solving the problem of uneven contribution of multi-scale features.

Slim-BiFPN takes lightweight deployment as the core goal, uses DWConv and GhostConv to reduce computational complexity, simplifies the BiFPN structure to form Slim-BiFPN, and embeds CBAM attention to focus on pest features (Xu et al., 2025). Its design focuses on “computational efficiency + target attention”, but the simplified feature fusion structure weakens the interaction between deep and shallow features, and it does not involve high-resolution detection layers, leading to limited performance in detecting ultra-small targets such as tiny olive fruits.

Lightweight Transformer combines Transformer’s global attention with CNN’s local feature extraction advantages, and adopts GhostNet’s lightweight idea to optimize the backbone (Xia et al., 2025). Its technical focus is “global-local feature integration”, but Transformer’s inherent computational complexity still restricts its real-time performance, and the lack of a dedicated cross-scale fusion module results in insufficient adaptation to multi-scale small targets in complex agricultural backgrounds.

YOLO-TinyFuse Ours integrates four key modules P2 layer, BiFPN, ModifiedNeck, decoupled detection head to form a “detail retention-fusion optimization-task specialization” collaborative framework. Compared with the above models, it not only retains shallow high-resolution features through the P2 layer similar to YOLOv8-p2 but also solves the problem of uneven multi-scale feature contribution through ModifiedNeck’s adaptive attention weighting making up for the deficiencies of YOLOv8-p2 and Slim-BiFPN. Meanwhile, the decoupled detection head realizes task-specific feature learning a design not involved in the other three models, and the lightweight structure ensures deployment feasibility comparable to Slim-BiFPN.

2. Performance and Scenario Adaptability Trade-offs

In terms of accuracy vs. lightweight balance, YOLOv8-p2 achieves an mAP50 of 91.9% with 7.39M parameters, showing high accuracy but relatively large model size, which is not conducive to edge device deployment. Slim-BiFPN reduces the parameter count to 1.0M, but its mAP performance on ultra-small targets is limited due to the lack of a P2 layer. The Lightweight Transformer has a mAP of only 90.7% on tea bud datasets, indicating poor adaptation to dense small targets. YOLO-TinyFuse achieves a balance with 2.96M parameters and high mAP50 87.76% for mangoes, 93.74% for apples, being 60% lighter than YOLOv8-p2 while maintaining comparable accuracy, and outperforming Slim-BiFPN and Lightweight Transformer in ultra-small and dense target scenarios.

Regarding scenario specialization vs. generalization, YOLOv8-p2 is optimized for UAV agricultural imagery, and Slim-BiFPN is specialized for tiny pest detection, both lacking cross-scenario adaptability. The Lightweight Transformer is designed for tea bud detection, with poor generalization to occluded targets. YOLO-TinyFuse targets universal small-object detection challenges detail loss, occlusion, complex backgrounds rather than crop-specific features, achieving stable performance across olive, wheat, cherry, mango, and apple detection mAP50 86.7%-95.8%, demonstrating superior generalization.

3. Core Advantage of YOLO-TinyFuse

The multi-module collaborative design of YOLO-TinyFuse addresses the limitations of single-module optimization in existing models: 1) The combination of P2 layer and BiFPN makes up for the “semantic-detail disconnection” in Slim-BiFPN and Lightweight Transformer; 2) ModifiedNeck’s adaptive weighting solves the problem of equal-weight fusion in YOLOv8-p2 and Slim-BiFPN; 3) The decoupled detection head eliminates the task interference in shared convolution architectures, which is not considered in the other three models. This structural synergy enables YOLO-TinyFuse to balance accuracy, lightweight, and generalization, making it more suitable for complex agricultural field environments requiring real-time edge deployment.

### 4.3 Limitations

Although the YOLO-TinyFuse model exhibits significant advantages in olive fruit detection tasks, it still has several limitations. Firstly, the definition of “real-time” in this study is that the model can be deployed on mainstream edge computing platforms or UAV-based systems with an inference speed meeting the practical application requirement of 15 frames per second (FPS). However, in olive orchard scenarios, if the model is deployed on low-end resource-constrained mobile or embedded devices such as low-power microcontrollers and entry-level single-board computers, its inference speed cannot meet the requirements for real-time deployment. When running on such devices, the model is prone to inference latency; when applied in environments with dense fruits or complex backgrounds, the frame rate will further decrease, leading to delayed detection results. Such inference latency and detection delay will not only affect the continuity and accuracy of target tracking but also reduce the detection coverage efficiency of large-scale olive orchard inspections, making it difficult to meet the practical demand for efficient detection in large-scale operation scenarios.

Secondly, the performance of the model during training is limited by the quality and diversity of the available dataset. While the current dataset includes challenging conditions such as low illumination, strong highlights, fruit occlusion and motion blur, it lacks scenarios involving fruit-surface lesions caused by diseases or pests. Consequently, the model’s robustness in adverse weather conditions and in the presence of abnormal fruit colouration or other atypical visual appearances remains insufficiently validated, and its applicability to a broader range of real-world scenarios cannot yet be fully ensured.

In addition to the aforementioned limitations, the model also has certain constraints and boundary conditions, which not only define its valid application scope but also reveal the intrinsic defects of its structural design. The model tends to fail under specific extreme scenarios, and heatmap visualization of the P2 layer and deep semantic layers further explains the root causes: when olive fruits are in the early fruiting stage and coexist with small impurities, the model easily misclassifies impurities as small fruits or misses tiny fruits entirely, as the visualization results show that the P2 layer cannot effectively distinguish fine-grained texture differences between targets with similar pixel sizes, leading to confused attention focus. In mixed-crop orchards with interplanted small-fruited plants, the feature fusion mechanism optimized for olives struggles to separate morphologically similar heterologous targets, and visual analysis of ModifiedNeck feature weighting indicates it overemphasizes shape features shared by similar targets, resulting in a sharp increase in false positives. Under extreme weather conditions such as heavy fog, dense haze, or intense backlighting, BiFPN fails to filter noise features, and the blurred visualization results of cross-scale feature transmission confirm invalid noise interference, causing significant degradation in detection accuracy. These failure cases, supported by visual evidence, delineate the model’s clear boundary conditions—it performs stably only for fruits without external impurities attached, in single-crop orchards, and under moderate environmental conditions. Meanwhile, the “black box” nature of feature transmission and weighting in the model leads to insufficient interpretability: when the fruit occlusion rate is high, visualization cannot clarify whether texture, shape, or color dominates the detection decision, making it difficult to diagnose the root causes of failures and hindering targeted optimization.

Furthermore, the model has limitations when faced with severe fruit occlusion. When olive fruits are heavily obscured or almost completely concealed by branches and foliage, the Recall rate drops significantly, making accurate identification and localisation difficult. This is because the current feature extraction and fusion mechanisms are unable to capture enough discriminative and semantically valid features in cases of extreme occlusion. This results in reduced reliability of detection for highly concealed targets.

## 5 Conclusion

In response to the challenges posed by complex orchard backgrounds, small fruit size, and frequent occlusion in the detection of olives, this study presents an enhanced version of the YOLO-TinyFuse model. The model achieves simultaneous advances in detection performance and lightweight design by integrating three key enhancements: the P2 high-resolution detection layer; the ModifiedNeck cross-scale fusion module; and the BiFPN bidirectional feature-aggregation mechanism. YOLO-TinyFuse improves recognition accuracy under leaf occlusion and heterogeneous illumination, and enhances hardware adaptability through its reduced parameter count. This delivers a detection framework that balances high Precision with computational efficiency.

Experimental results show that YOLO-TinyFuse achieves an mAP50 of 0.923 and an F1 score of 0.869 on the olive fruit detection test set with only 2.96 million parameters. Not only does the model markedly outperform mainstream detectors such as DetrR50-Dc5, DetrR50, YOLOv5n, YOLOv9t and YOLOv11n, it also substantially reduces computational redundancy due to its compact architecture. This lightweight design enables direct deployment on edge-computing platforms, such as the Raspberry Pi 4B, and on unmanned aerial vehicles. It overcomes the conventional constraint that achieving high accuracy requires a high computational cost. In terms of practical applicability, YOLO-TinyFuse closely aligns with the full-chain operational requirements of the olive industry. Furthermore, its technical framework is highly reusable, facilitating rapid adaptation to small-object tasks involving wheat, cherries, and bees, as well as crops with high occlusion rates, such as mangoes and apples. This generalisation capability supports the transition from single-crop-specific detection to unified, multi-crop intelligent solutions, thereby reducing costs and improving efficiency in agricultural production.

We recognise the current limitations of the model in terms of inference speed, training data coverage, and detection of highly occluded targets. We regard these aspects as key areas for future improvement. Subsequent work will explore pruning, quantisation and other lightweight optimisation techniques to reduce model parameters and computational cost further, while integrating Transformer-based attention mechanisms to enhance feature capture efficiency. In parallel, the dataset will be expanded to include more challenging scenarios, such as fruit affected by pests or diseases, and severe occlusion. Techniques such as targeted data augmentation, fine-grained annotation and collaborative training will be employed to improve the model’s adaptability to adverse weather conditions and atypical fruit appearances. Furthermore, we will refine the feature-fusion and feature-extraction mechanisms to strengthen the model’s ability to identify and localise heavily occluded fruit. This will enhance its overall practical utility and generalisation capacity.

## Data Availability

The raw data supporting the conclusions of this article will be made available by the authors, without undue reservation.
